# Unravelling the Origin of Water’s Thermal Conductivity Maximum: Compressibility, Tetrahedrality and Nuclear Quantum Effects

**DOI:** 10.1021/jacs.4c12898

**Published:** 2025-11-04

**Authors:** Oliver R. Gittus, Fernando Bresme

**Affiliations:** Department of Chemistry, Molecular Sciences Research Hub, 4615 Imperial College London, London W12 0BZ, U.K.

## Abstract

Water is arguably the most important liquid on Earth. Consequently, its anomalous properties have been intensely investigated for over 50 years. However, water’s thermal conductivity maximum (TCM) remains hitherto unexplained. Beyond its substantial fundamental interest, this problem is critical because many natural (e.g., climate regulation), industrial and chemical processes in which water appears as solvent at near-standard conditions correspond to the anomalous heat transport regime of water. We use all-atom and minimal coarse-grained models to isolate the TCM’s thermodynamic fingerprint, and subsequently demonstrate its thermodynamic and microscopic origin: (1) the depopulation of librational modes due to nuclear quantum effects and (2) the balance of two interconverting molecular arrangements, the high density and low density liquid states, that coexist in water. We systematically investigate tetrahedral liquids modeled with Stillinger-Weber potentials, which allows the interpolation between simple liquids and low coordination materials such as carbon. We show that the TCM is not exclusive to water, but an anomalous behavior shared by pure liquids with intermediate tetrahedrality. Our work provides a thermodynamic explanation for the TCM of water and tetrahedral liquids in general.

## Graphical abstract

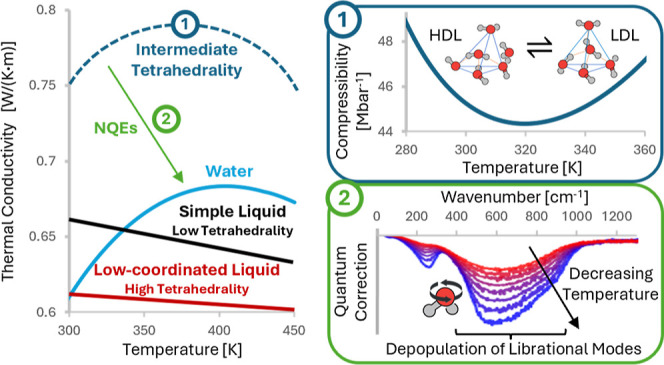

## Introduction

Water plays a central role in our lives: it is ubiquitous in nature and industry, necessary for life on Earth, and by definition hosts the entirety of aqueous chemistry. Furthermore, water is a widely used solvent in many chemical processes, earning it the epithet of “universal solvent”. Despite its relatively simple molecular geometry, a triatomic molecule with *C*_2v_ symmetry, water is extremely complex in its condensed phases: it has an enormously rich phase diagram with different types of ices, amorphous phases, and a supercooled liquid state in which many anomalous properties are enhanced.^1^ Liquid water at ambient conditions also possesses many anomalous properties, most notably a density maximum at 4 °C (277.15 K) and (constant) atmospheric pressure, and a solid phase that is less dense than the liquid (i.e., ice floats in liquid water). Its thermodynamic response functions, such as the isothermal compressibility β_T_, thermal expansion coefficient α_P_ and isobaric heat capacity *C*_P_, also show anomalous behaviors.

Beginning in the 19th century with the mixture models of Whiting and Röntgen,^2,3^ these anomalies can be explained when water is viewed as a mixture of two interconvertible molecular arrangements, often referred to as the high-density liquid (HDL) and low-density liquid (LDL) structures. Within this two-state picture, several qualitatively distinct scenarios have been proposed: the stability limit conjecture,^4^ the liquid–liquid critical point (LLCP),^5^ the critical-point-free^6^ and the singularity-free^7^ scenarios. State-of-the-art molecular simulations favor the “second critical point” scenario,^8,9^ while mounting indirect experimental evidence is consistent with, but does not yet conclusively prove, the existence of the LLCP (see ref 1 and the references therein). These thermodynamic scenarios explain water’s thermodynamic anomalies, and while conceptual bridges have been built for diffusivity and viscosity,^1,10–17^ water’s heat transport anomalies and the associated microscopic mechanisms remain unexplained.

At constant pressure the thermal conductivity (TC) of liquid water increases with temperature until it reaches a maximum at 404 K (10 bar),^18^ then decreases upon further heating until the boiling point is reached. In contrast, the TC of a simple liquid monotonically decreases with increasing temperature primarily due to the corresponding decrease in density. Thus, water’s thermal conductivity maximum (TCM) and subsequent decrease upon cooling are anomalous properties. The temperature of the TCM increases with pressure; all natural and industrial processes that occur at near-standard conditions therefore correspond to the anomalous heat transport regime of water. Despite its significance, the physical origin of water’s TCM remains an open question.

Theoretically predicting the TC of liquids is a challenging problem. In arguably the earliest attempt (1923), Bridgman connected the TC of a liquid to its isentropic speed of sound. He imagined liquid molecules arranged in a cubic lattice, with the internal energy difference due to the temperature gradient being “handed down a row of molecules at a rate determined by the speed of sound”.^19^ This was the first in a family of quasi-lattice (QL) models that assume liquid molecules oscillate about fixed points in a solid-like lattice on the time scale of heat transport, and exchange energy via nearest-neighbor collisions.^20–24^ When the characteristic frequency of energy exchange is identified with the acoustic spectrum, these models give a TC of the form λ_QL_ ∝ δ^–2^ *c*, where δ is the average distance between molecules and *c* is the speed of sound.^23,24^ While these models may give accurate predictions for specific liquids at specific thermodynamic conditions, they cannot in general predict the TC of liquids.^20,22,23,25,26^ However, we will show that when used together with simulations, they provide insight into the origins of water’s TCM.

We consider one of simplest quasi-lattice models: the Bridgman equation empirically corrected for polyatomic molecules, λ_B_ = 2.8*k*_B_δ^–2^ *c*_S_, which using the thermodynamic relations $c_{S}^{2}={\left(\rho \beta_{S}\right)}^{-1}$ and β_T_/β_S_ = *C*_P_/*C*_V_ = γ, can be written as

1 $$\lambda_{B}=2.8k_{B}{\left(\frac{{\rho \gamma }^{3}}{M^{4}\beta_{T}^{3}}\right)}^{1/6}$$

where ρ, *M*, β_T_ (β_S_) and γ are the density, molecular mass, isothermal (isentropic) compressibility and adiabatic index, respectively. *C*_P_ and *C*_V_ are the isobaric and isochoric heat capacities, respectively. Modern interpretations of the Bridgman equation identify the preceding factor, set here to 2.8 *k*_B_, as the heat capacity of the vibrational modes that transport heat.^22–24^ The Bridgman equation can also be recovered within phonon models of liquids when the group velocity of heat carrying modes is approximated as *c*_S_ (a very good approximation for simple liquids such as argon).^23^ In the case of water, ρ­(*T*) and γ­(*T*) both increase monotonically in the temperature range where the extrema in λ_B_, *c*_S_, β_S_ and β_T_ occur. Thus, all four extrema share the same phenomenological origin, i.e., if one did not exist, then they all would not exist, and the effect of ρ­(*T*) and γ­(*T*) is to shift the temperature *T*_ex_ of the extrema. Thus, the Bridgman equation, along with other quasi-lattice models, provides a possible thermodynamic explanation for the TCM.

The Bridgman equation is surprisingly accurate for water (and some other liquids) at near-standard conditions.^18,27^ It accurately reproduces the experimental TC down to 0 °C (273.15 K) at 1 bar (the limit of validity of the experimental IAPWS-2011 correlation^18^) and extrapolations have been used to predict the anomalous behavior (a TC minimum) of supercooled water (see Section 2.1 in the Supporting Information for a more in depth discussion of the Bridgman equation).^28^ However, eq 1 breaks down at high temperatures.^18,27^ Furthermore, advancing our results below, the magnitude of the Bridgman equation does not hold for molecular force fields of water even at near-standard conditions: it underestimates the TC by ∼10–40% (overestimates by ∼180% for mW) at 300 K. A popular approach is to replace the factor of 2.8 with a fitted coefficient, resulting in accurate predictions for a wide array of fluids, especially for monatomic and diatomic liquids.^25^ The fitted coefficient is ∼1 for monatomic fluids and generally increases with molecular complexity;^25^ for water it is ∼3, and ∼1.8 at extreme conditions (1000–2000 K and 1.0–1.9 g cm^–3^; up to 22 GPa).^25,26^ This demonstrates that the TC is highly correlated with the speed of sound, and supports the use of the Bridgman equation to predict the position and existence of the TCM, which do not change when scaling eq 1 by a constant. Even so, while eq 1 reproduces the TCM, it is shifted by $T_{\max\left(\lambda_{B}\right)}-T_{\max\left(\lambda \right)}∼-70$ K at near-standard pressure, and $\left|T_{\max\left(\lambda_{B}\right)}-T_{\max\left(\lambda \right)}\right|$ increases with pressure.^18^

The question remains: to what extent is the TCM connected with the β_T_ minimum? This hypothesis has eluded investigation by simulation studies because it is difficult to build an accurate model of water that does not reproduce the compressibility minimum, which reflects crucial aspects of the orientational correlations and tetrahedral order in liquid water. Furthermore, owing to the microscopic formulation of the heat flux^29–31^ and the presence of coupling effects,^32,33^ it is still challenging to calculate TC from simulations, and the TCM has seldom been reported. Advancing our discussion below, we identify seven water models that reproduce the TCM, and crucially, two that do not. Through the analysis of these different models, we identify three factors contributing to the observation of the TCM: the compressibility via thermodynamic considerations, tetrahedrality and nuclear quantum effects.

## Methods

We perform extensive equilibrium and nonequilibrium molecular dynamics simulations for a diversity of water models: the rigid nonpolarizable force fields TIP3P,^34^ SPC,^35^ SPC/E,^36^ TIP4P/2005 ^37^ and TIP5P;^38^ the flexible model TIP4P/2005f;^39^ the flexible, polarizable and reactive force fields (ReaxFF), water-2017 ^40^ and CHON-2017_weak;^41^ and the highly coarse-grained monatomic water “mW” model^42^ together with related Stillinger-Weber (SW) potentials.^43^ Thermodynamic response functions were calculated from *NPT* simulations using the fluctuation relations and the equation of state. The TCs were calculated from boundary-driven nonequilibrium molecular dynamics (NEMD) simulations using Fourier’s Law, *J*_q_ =–λ∇*T*, where *J*_q_ is the heat flux and ∇*T* is the local temperature gradient. As demonstrated in our previous work,^44^ the temperature gradients used here are well within the linear regime: ∇*T* < 2, 8, and 11 K nm^–1^ for the mW/SW, empirical and ReaxFF force fields, respectively. We note that the computation of TC via NEMD includes all possible coupling effects; in the case of polar fluids such as water this includes the coupling between heat and polarization fluxes, which decreases the TC.^32,33,45^ We converge our TC values with respect to the NEMD simulation cell size. Statistical uncertainties reported herein correspond to the 95% confidence interval and the uncertainties of fit parameters (e.g., *T*_ex_ obtained by fitting cubic functions to the region close to the minimum/maximum) were estimated by parametric bootstrapping. Further methodological details are given in the Supporting Information.

## Results and Discussion

We show in Figure 1a­(iii) the TC of the water models at constant pressure, alongside the experimental values. We target the 10 bar isobar since at 1 bar the TCM occurs above the boiling point and experimental data for superheated water is not available. For each force field, a single λ­(*P*, *T*) point is calculated from each simulation, and the TC at 10 bar is calculated by interpolating λ­(*P*) at a given *T* (see Section 2.7.1 in the Supporting Information). Consistent with the growing body of work^44,46–63^ demonstrating that empirical atomistic force fields typically overestimate the TC by ∼10–50% at temperatures/pressures near 300 K and 1 bar, the atomistic force fields reported here systematically overestimate λ by 30–50% at near-standard conditions. It is evident in Figure 1a that the temperature dependence of λ is correlated with that of λ_B_ and β_T_. For example, SPC/E exhibits shallow extrema in λ, λ_B_ and β_T_. Similarly, comparing the reactive force fields, water-2017 has steeper gradients than CHON-2017_weak for all three properties.

**Figure 1:**
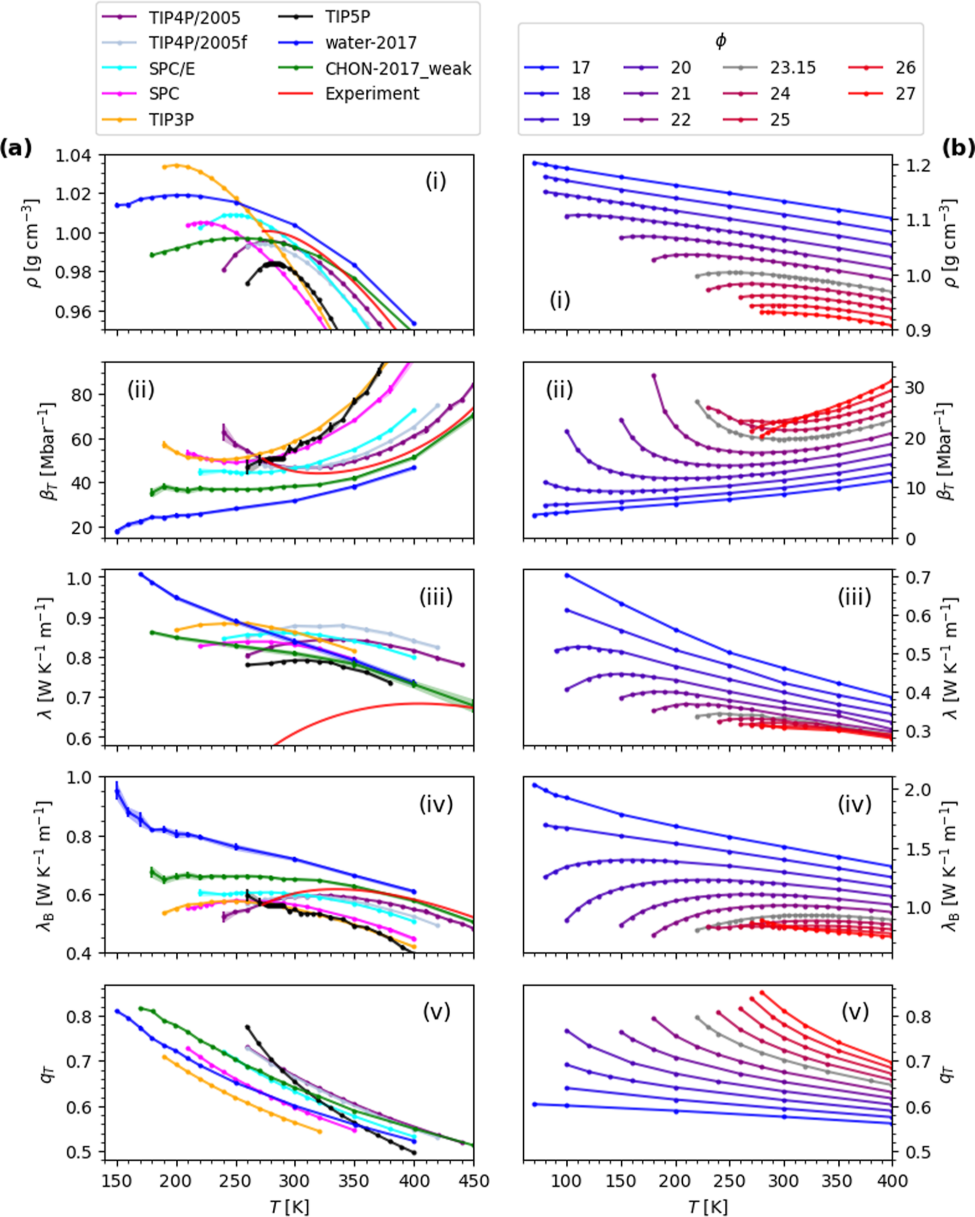
Thermophysical and structural properties of (a) atomistic force fields of water and (b) SW potentials as a function of temperature *T* at constant pressure: the (i) density ρ, (ii) isothermal compressibility β_T_, (iii) thermal conductivity λ, (iv) Bridgman thermal conductivity λ_B_ and (v) the orientational tetrahedral order parameter *q*_T_. In (b), ϕ represents the strength of the three-body interactions that promote tetrahedral order. Data corresponds to ∼10 bar. Experimental data are from, or calculated from, refs 18 and 27 .

The TCM can be inferred for TIP4P/2005, SPC/E and MCFM from previous NEMD simulation studies,^56,57^ but quantitative estimates of *T*_max(λ)_ were not reported. Studies using the Green–Kubo (GK) method have explicitly shown TCM at near-standard pressures (exact pressure conditions were not specified) for TIP4P/2005 (*T*_max(λ)_ ∼ 400 K), SPC/E (*T*_max(λ)_ ∼ 400 K) and TIP4P (*T*_max(λ)_ ∼ 350 K).^60,61^ However, these studies report λ values as low as 0.2 W K^–1^ m^–1^ at 240–250 K, which is inconsistent with the 0.8–0.9 W K^–1^ m^–1^ from other^64,65^ GK predictions using the same models. Thus, to the best of our knowledge, we report the first reliable quantitative predictions of *T*_max(λ)_ using empirical force fields.

The temperatures at which the extrema occur are shown in Figure 2 (see Table 2 in the Supporting Information for numerical data). TIP4P/2005, TIP4P/2005f, SPC/E, SPC and TIP3P reproduce the TCM. They also predict the correct order of thermodynamic anomalies, $T_{\max\left(\rho \right)}<T_{\min\left(\beta_{T}\right)}<T_{\max\left(\lambda_{B}\right)}<T_{\min\left(\beta_{S}\right)}<T_{\max\left(c_{S}\right)}$ , which is expected since $T_{\max\left(\rho \right)}<T_{\min\left(\beta_{T}\right)}<T_{\min\left(\beta_{S}\right)}<T_{\max\left(c_{S}\right)}$ is a thermodynamic necessity for (∂*T*_max(ρ)_/∂*P*) < 0,^7,66^ and $\alpha_{P}>0\Rightarrow T_{\max\left(\lambda_{B}\right)}<T_{\min\left(\beta_{S}\right)}$ . Regarding the position of the TCM, TIP4P/2005, TIP4P/2005f and SPC/E correctly predict $T_{\max\left(\lambda \right)}>T_{\max\left(c_{S}\right)}$ (noting the overlap of uncertainties for TIP4P/2005 and TIP4P/2005f), while SPC and TIP3P predict $T_{\min\left(\beta_{T}\right)}<T_{\max\left(\lambda \right)}∼T_{\max\left(\lambda_{B}\right)}≲T_{\max\left(c_{S}\right)}$ . Interestingly, the two models, water-2017 and CHON-2017_weak, that fail to reproduce the TCM also fail to reproduce the extrema in the thermodynamic properties β_T_, β_S_, *c*_S_ and λ_B_. Our simulation results would therefore support the hypothesis that the TCM arises from the compressibility minimum. However, TIP5P does reproduce the TCM, although with a weak temperature dependence at lower temperatures, but not the four thermodynamic extrema.

**Figure 2:**
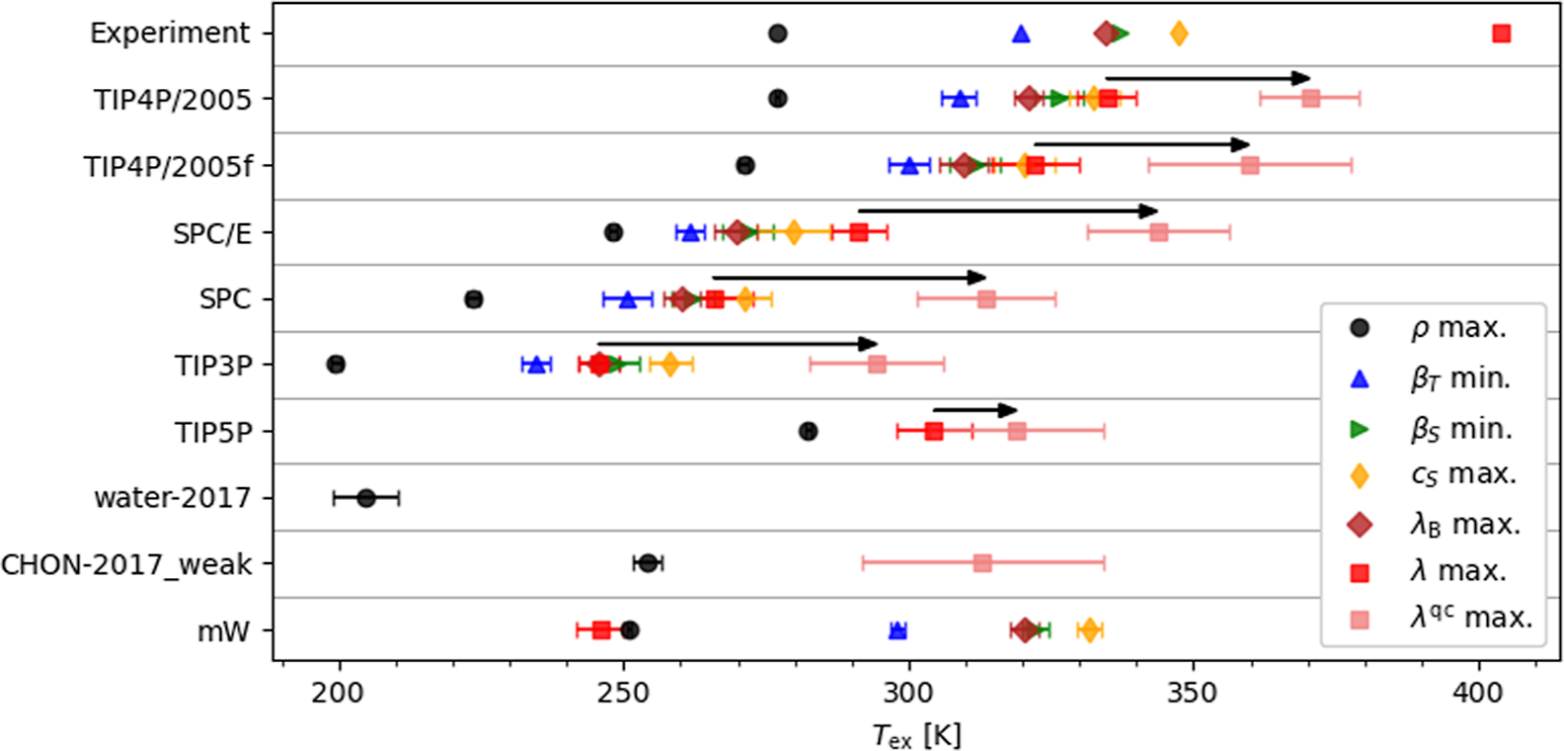
Temperature *T*_ex_ at which extrema in thermophysical properties occur for the force fields of water at 10 bar. The arrows indicate the temperature shift in the TCM, from *T*_max(λ)_ to $T_{\max\left(\lambda^{qc}\right)}$ , due to nuclear quantum effects. Experimental data are from, or calculated from, refs 18 and 27 .

We show in the Supporting Information that for TIP5P the pressure of the maximum *T*_max(ρ)_(*P*) is *P*_max(TMD)_ = (250 ± 90) bar. At this pressure, the extrema in β_T_, β_S_, *c*_S_ and λ_B_ will appear at the same temperature as the density maximum, *T*_ex_ = (282.8 ± 0.3) K, then move to higher temperatures as *P* is increased, diverging from *T*_max(ρ)_ which decreases.^67,68^ Thus, the Bridgman equation predicts the TCM at only ∼200–300 bar higher, corresponding to a density difference of ∼0.01 g cm^–3^, supporting the view that the TCM is correlated with these thermodynamic anomalies. We will show later that investigating tetrahedral liquids modeled using the mW/SW potentials gives insight into the behavior of TIP5P.

The empirical atomistic force fields underestimate *T*_max(λ)_ by ∼70–160 K. Our simulations were carried out according to classical nuclear dynamics, i.e., nuclear quantum effects (NQEs) are not explicitly accounted for. However, NQEs are implicitly included, to some extent, in classical models fit to experimental data. While this is not the case for ReaxFF models if they are parametrized using only ab initio data, both water-2017 and CHON-2017_weak were parametrized using DFT and experimental data, which notably include experimental liquid densities.^40,41^ Recently, a machine-learned (neuroevolution) potential (MLP) trained at the quantum-mechanical DFT level with the SCAN functional predicted the TCM at 30 bar only when corrected for NQEs.^69^ However, deep neural network potentials, one also trained on SCAN and the other on PBE, reproduced the TCM without NQEs.^70^ The discrepancy between the two MLP-SCAN results may in part be attributed to the use of experimental densities along an “ambient pressure” isobar in ref 70, as opposed to ρ­(*T*) of the model. Our simulations using empirical force fields show that the explicit incorporation of NQEs is not required to observe the TCM. Furthermore, advancing its introduction below, the purely classical mW model possesses the TCM, despite not including light atoms (hydrogen), which are primarily responsible for NQEs. The mW results demonstrate that NQEs are not strictly necessary for the existence of the TCM.

To investigate the impact of NQEs on the TCM we quantum-correct our TC values through the isobaric heat capacity *C*_P_, which is related to the TC via λ = ρ*C*_P_ *D*_T_, where *D*_T_ is the thermal diffusivity. The quantum-corrected TC, λ^qc^, is given by

2 $$\lambda^{qc}=\rho C_{P}^{qc}D_{T}=\left(C_{P}^{qc}/C_{P}\right)\lambda =\Delta^{qc}\lambda$$

This approach relies on the fact that water’s heat capacity is “a signature of nuclear quantum effects” ^71,72^ and is greatly overestimated by classical models even at high temperatures (see Figure S4 in the Supporting Information), while *D*_T_ is much less sensitive to NQEs.^69,73^ An analogous heat capacity scaling approach predicted TCs in good agreement with experiment for liquid para-hydrogen and helium at the CMD-PIMD level of theory.^73^ We employ the frequency domain method of Berens^74^ to quantum-correct *C*_P_ (see Section 2.5 in the Supporting Information). We note that the quantum-corrected *C*_P_ values for TIP4P/2005 and TIP4P/2005f are in excellent agreement (≲4% from 273 to 440 K) with experiment (see Figure S4 in the Supporting Information).

The quantum correction decreases the TCs of the atomistic force fields, bringing them into much better agreement with experiment (Figure 3a). As shown in Figure 3b, the quantum correction is significantly larger for the flexible force fields. Classically, water’s intramolecular vibrational modes are relatively small but significant heat carriers,^69^ as demonstrated by the systematic ∼3–6% increase in TC from TIP4P/2005 to TIP4P/2005f (Figure 1a­(iii)). Quantum mechanically, these vibrational modes are not populated at ambient temperatures. This is reflected in our quantum correction to *C*_P_, in which the contribution of intramolecular vibrations are almost completely suppressed (>95% even at the highest temperatures, 400–450 K), consistent with the similar quantum correction to the potential–potential part of the spectral thermal conductivity of MLP-SCAN in ref 69. Overall, the intramolecular contribution Δ_intra_^qc^ is large, between 32 and 35% of the total Δ^qc^ at 300 K and ∼20–50% depending on the temperature (see the Supporting Information, Figure S5).

**Figure 3:**
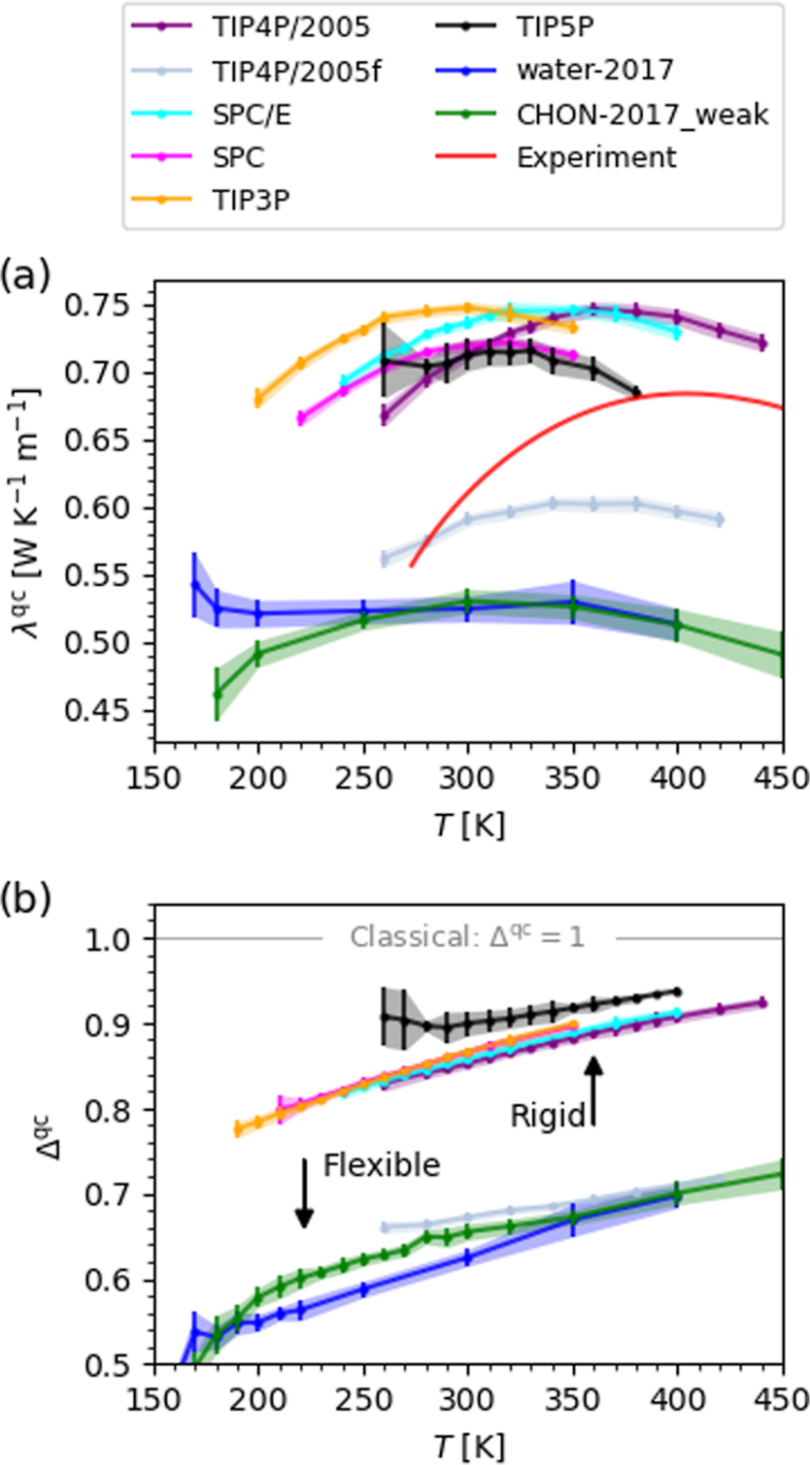
Effect of nuclear quantum effects on the thermal conductivity of selected atomistic force fields. (a) The quantum-corrected thermal conductivity λ^qc^ and (b) quantum-correction factor Δ^qc^ as a function of the temperature *T*. Data corresponds to 10 bar.

Returning to *T*_max(λ)_, it is the temperature dependence of Δ^qc^ that has an effect. Δ^qc^ increases monotonically with temperature, which shifts the TCM to higher temperature, in better agreement with experiment (Figure 2). For the flexible (rigid) force fields, the temperature dependence of Δ^qc^ comes almost (exactly) entirely from the intermolecular contribution Δ_inter_^qc^ (see the Supporting Information, Figure S5). The majority of Δ_intra_^qc^ and its temperature dependence arises from librational modes (see the Supporting Information, Figure S6) and we therefore identify the depopulation of librational modes as the primary molecular mechanism for the increase in *T*_max(λ)_ due to NQEs. (This does not imply that librations are the primary contributors to the total TC: lower frequency modes are also significant, especially at lower temperatures.^46,65,69^ ) Interestingly, the quantum correction induces the TCM in CHON-2017_weak, as is the case for MLP-SCAN in ref 69.

We turn to the mW/SW models to strengthen the connection between the TCM, the thermodynamic extrema and the microscopic two-state picture of water. The mW model underestimates the TC of water by 45% at 300 K, and is ∼60% lower than the atomistic force fields, but crucially it predicts a TCM (see Figure 1b­(iii)). The TCM is also shifted to a lower temperature in the order of extrema (see Figure 2). Decompositions of the microscopic heat flux in atomistic simulations show that Coulomb interactions,^75^ rotational intermolecular energy transfer,^46^ and the heat flux carried by the hydrogen atoms^56^ are major contributors to water’s TC. The mW model lacks all these mechanisms of heat transfer, explaining its lower TC. Indeed, the mW model does not aim to accurately incorporate all the molecular details of water, but rather to capture its phenomenology as a tetrahedral liquid.

The mW model is a paramaterization of the SW potential for water. The SW potential has the form $V_{SW}=V_{2}+\phi V_{3}$ , where $V_{2}$ and $V_{3}$ are the potential energy contributions of the two- and three-body terms, respectively. $V_{3}$ imposes an energetic penalty for deviations from the tetrahedral angle cos θ = −1/3 (θ ≈ 109.5°) between triplets of particles. Increasing ϕ therefore increases the degree of tetrahedral order of the liquid. To show this, we compute the orientational tetrahedral order parameter $q_{T}=1-\left(3/8\right)\sum_{j-1}^{3}\sum_{k=j+1}^{4}{\left(\cos\psi_{jk}+1/3\right)}^{2}$ ,^76^ where ψ_*jk*_ is the angle formed by the lines joining the central particle (water oxygen atom for the atomistic force fields) and its nearest neighbors *j* and *k*. *q*_T_ measures the local tetrahedral order taking into account the four nearest neighbors and varies from 0 for an ideal gas to 1 for a regular tetrahedron. Figure 1b­(v) shows that *q*_T_ increases with ϕ at a given temperature.

We systematically investigate the effect of tetrahedrality on the TCM by varying ϕ. We show in Figure 1b ρ, β_T_, λ and λ_B_ for 17 ≤ ϕ ≤ 27 at 10 bar. At constant temperature and pressure, the TC generally decreases with increasing ϕ due to the corresponding decrease in density and speed of sound. The behavior of λ_B_(*T*; ϕ) mirrors that of λ­(*T*; ϕ), justifying the interpretation with eq 1. We show in Figure 4 the temperature *T*_ex_ of the TCM and other extrema for the SW model. *T*_ex_ features a maximum at ϕ ∼ 24 (close to ϕ = 23.15 corresponding to water) for β_T_, β_S_, *c*_S_ and λ_B_, while *T*_max(ρ)_ and *T*_max(λ)_ increase monotonically with ϕ.

**Figure 4:**
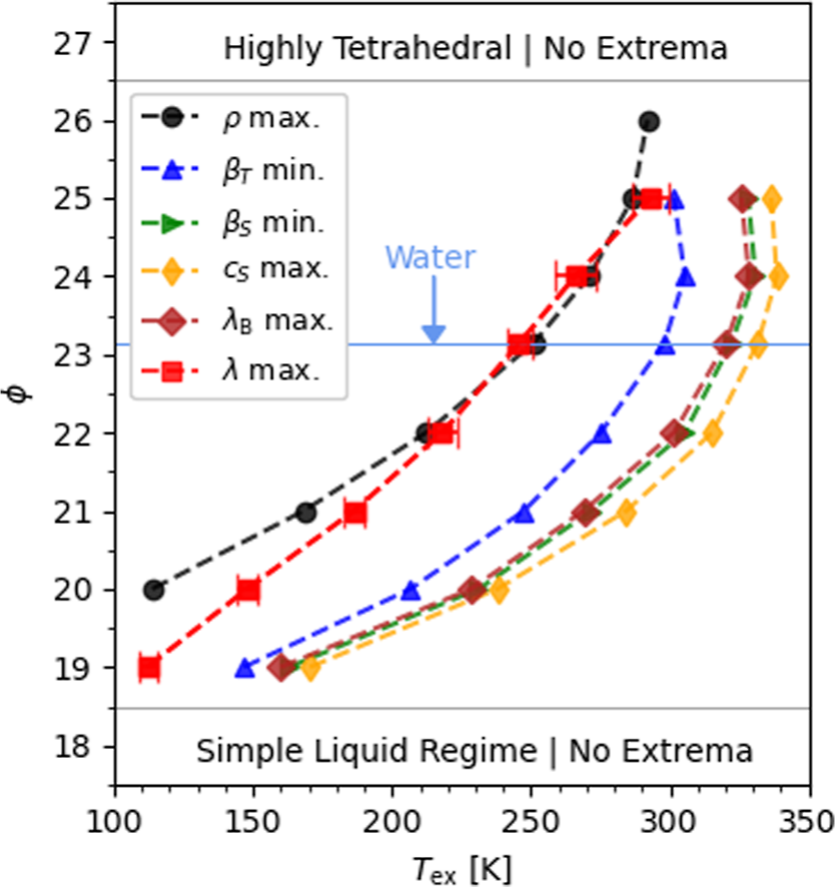
Temperature *T*_ex_ at which extrema in thermophysical properties occur for the SW potentials at 10 bar. The “tetrahedrality” parameter ϕ controls the strength of the interaction energy that penalizes deviations from the tetrahedral angle θ ≈ 109.5° between triplets of molecules.

Decreasing ϕ from the mW model (ϕ = 23.15) to ϕ = 17 interpolates between liquid water and a simple liquid. First the density maximum is lost at 19 ≤ ϕ < 20, followed by the TCM together with the extrema in β_T_, β_S_, *c*_S_ and λ_B_ at 18 ≤ ϕ < 19, reaffirming the connection between the TCM and the compressibility minimum. Thus, the SW model transitions from tetrahedral liquid behavior at ϕ ∼ 20 (coordination number *n*_c_ ∼ 8.5) to that of a simple fluid at ϕ ∼ 18 (*n*_c_ ∼ 10). In addition to the increasing *n*_c_ with decreasing ϕ, we observe a less prominent peak in the radial distribution function at ∼4.5 Å (see Supporting Information Figures S15 and S16) indicative of weaker tetrahedral order. This peak disappears at ϕ ∼ 18, marking the onset of the simple liquid regime. Analogously, increasing ϕ from the mW model (*n*_c_ ∼ 5.0) to ϕ = 27 (*n*_c_ ∼ 4.1–4.5) interpolates between the behavior of water and that of highly tetrahedral materials such as carbon (ϕ = 26.2 ^77^). In this case, the TCM disappears along with the extrema in β_T_, β_S_, *c*_S_ and λ_B_ at 25 < ϕ ≤ 26, before the density maximum is lost at 26 < ϕ ≤ 27. This once again reaffirms the connection between the TCM and the compressibility minimum. Thus, the TCM exists in a “Goldilocks Zone” of tetrahedrality, 18 < ϕ < 27, and disappears with the compressibility minimum at lower/higher ϕ.

Returning to TIP5P, which is known to feature higher tetrahedrality compared to other water models and experiment,^38,72,78^ the steeper gradient in *q*_T_(*T*) compared to the other atomistic water models (Figure 1a­(v)) is the trend observed when increasing ϕ in the SW potential (Figure 1b­(v)). In line with this, TIP5P features the density maximum at 10 bar but not the other thermodynamic anomalies, which is the scenario for ϕ = 25.

To establish the connection with the two-state picture, we show in Figure S7 in the Supporting Information the probability density functions *f* of *q*_T_ for the SW potentials at different temperatures. Increasing temperature at intermediate values of ϕ, *f*(*q*_T_) transitions from a single high-*q*_T_ peak corresponding to the tetrahedral LDL structure, to a bimodal distribution with a second peak at lower *q*_T_ indicative of a significant fraction of the more disordered HDL structure. In contrast, in the highly tetrahedral regime (ϕ = 27) where the anomalies disappear, the second low-*q*_T_ peak does not develop past a shoulder, even at 400 K. This corresponds to a smaller increase in the HDL fraction with temperature and/or that the HDL is more tetrahedral with a smaller density difference between the two states. We note that the larger fraction of HDL at lower ϕ is reflected in the overall increase in ρ­(ϕ; *T*) with decreasing ϕ. In the simple liquid regime, we observe broad distributions that are weakly temperature dependent, indicative of small changes in the fractions of the two states. Indeed, at ϕ = 17, *f*(*q*_T_) is single-peaked at a low *q*_T_ ≈ 0.5, corresponding to HDL, over the large 100–400 K temperature range. Thus, the TCM along with the thermodynamic anomalies arise due to sufficiently large changes in the HDL/LDL fraction as temperature is increased.

## Conclusion

In conclusion, using a wide range of water force fields we uncover the microscopic mechanisms that determine the maximum in the thermal conductivity of water. By examining why models succeed or fail to reproduce the TCM, we put forward two potential origins for the TCM that are not mutually exclusive: (1) it arises due to nuclear quantum effects and (2) it arises from thermodynamic considerations, via the compressibility minimum, from the balance of two distinct molecular arrangements in water, the HDL and LDL states.

Regarding (1), NQEs, acting primarily through the heat capacity, reduce the TC more at lower temperatures. In strongly quantum liquids, namely liquid para-hydrogen which possesses a TCM at ∼22 K along the saturation line, this can cause the monotonic decrease in TC expected for classical simple liquids to develop into a maximum.^73^ This would be surprising for water because the TCM is a high-temperature anomaly, occurring at ∼400 K at near-standard pressures at which NQEs are expected to be weak. Nevertheless, this is the case for the CHON-2017_weak and MLP-SCAN^69^ force fields. In models that already possess the TCM, including the accurate TIP4P/2005 and TIP4P/2005f force fields, correcting for NQEs increases *T*_max(λ)_. We identify the depopulation of librational modes as the primary microscopic mechanism responsible for the shift or appearance of the TCM due to NQEs. However, the purely classical mW/SW models possess the TCM, suggesting that NQEs are not strictly necessary for its existence. Furthermore, we show that NQEs reduce the TC of empirical water models and bring the value into better agreement with experimental data. This partly explains the near-universal overestimation of the TC by empirical force fields of water at near-standard temperature and pressure conditions.^44,46–63^

Regarding thermodynamic considerations (2), motivated by the Bridgman eq (eq 1), we show that the TCM is highly correlated with water’s compressibility minimum. Physically, this correlation stems from identifying low-frequency intermolecular vibrational modes as the primary heat carriers in liquids and approximating their “group velocity” by the speed of sound.^20–24^ The β_T_ minimum has been studied extensively, and microscopic explanations have been proposed by the now widely accepted two-state models in which two structural motifs coexist in water: a low-density state, stabilized enthalpically through tetrahedral hydrogen bonding, and a high-density state, stabilized entropically by its greater configurational disorder.^79–84^ An increased concentration of tetrahedral structures (increasing β_T_) upon cooling competes with the effect of increasing density (decreasing β_T_), leading to a minimum. Thus, we additionally provide a microscopic explanation for the TCM by connecting it with the β_T_ minimum. Indeed, using SW potentials for tetrahedral liquids, we show that a sufficiently large change in HLD/LDL fraction upon heating/cooling is required for the thermophysical anomalies to appear.

Using SW potentials for tetrahedral liquids, we investigate the TCM as a function of tetrahedrality, interpolating between the behavior of simple liquids and highly tetrahedral materials such as carbon. We show that the TCM vanishes alongside the compressibility minimum at both low and high tetrahedrality. Our results indicate that the TCM in real water exists in a “Goldilocks Zone” of tetrahedrality, arising from the balance between enthalpy and entropy in the liquid. Specifically, at intermediate tetrahedrality, such as that characteristic of water, structural fluctuations between the HDL and LDL states give rise to the thermophysical anomalies. At sufficiently low or high tetrahedralities, one of these states dominates and the TCM is lost. We therefore provide insight into the microscopic mechanism controlling the anomalous thermal transport in water. Our analysis of the Stillinger-Webber model suggests that this thermal transport anomaly can be controlled by tuning three-body interactions, and hence that the TCM is not exclusive to water, but a general physical behavior shared by low-coordination liquids of intermediate tetrahedrality.

## Supplementary Material



## Notes

Supporting Information includes simulation details for equilibrium and nonequilibrium MD, additional discussion on the Bridgman equation, thermodynamic response functions, density and structural analyses, quantum corrections to heat capacity, thermal conductivity data dependence with simulation cell size, and radial distribution functions. Tables with numerical data are provided (PDF)
