# Supplementary material for: Unravelling the Origin of Water’s Thermal Conductivity Maximum: Compressibility, Tetrahedrality and Nuclear Quantum Effects
Source: J Am Chem Soc. 2025 Nov 4;147(46):42175–83. doi: 10.1021/jacs.4c12898 (PMC12636029; doi:10.1021/jacs.4c12898)
Supplement: Supplementary file 1 [file ja4c12898_si_001.pdf]

Supporting Information

Unravelling the Origin of Water's Thermal Conductivity Maximum: Compressibility, Tetrahedrality and Nuclear Quantum Effects

Oliver R. Gittus and Fernando Bresme

Department of Chemistry, Molecular Sciences Research Hub,
Imperial College London,
London W12 0BZ, United Kingdom.

Contents

1 Simulation details **3**

1.1 Non-equilibrium molecular dynamics simulations 3

1.2 Equilibrium simulations 5

2 Additional results, data and discussion **6**

2.1 Additional discussion: the Bridgman equation in supercooled water 6

2.2 Numerical data for T_{ex} 7

2.3 Thermodynamic response functions 8

2.4 Density maximum of TIP5P 9

2.5 Quantum correction to the heat capacity 10

2.6 Orientational tetrahedral order parameter for the SW potentials 13

2.7 Thermal conductivity 14

2.7.1 Interpolating to P =10 bar 14

2.7.2 Finite-size effects and convergence 21

2.7.3 Thermal conductivities obtained using very large NEMD simulation cells 28

2.8 Radial distribution functions 31

1 Simulation details

We performed molecular dynamics (MD) simulations of water modelled using a diversity of molecular force fields (see the main text). All simulations were carried out using LAMMPS[1] (v. 7Aug2019). The equations of motion were integrated using the velocity Verlet algorithm, except for the 5-site rigid model TIP5P, for which the method of quaternions[2] for rigid bodies was used. For the rigid models (except TIP5P), the RATTLE[3] algorithm was used to maintain the holonomic constraints, except for the NPT simulations where SHAKE[4] was used. The following timesteps were used: $\delta t = 1$ fs for the rigid non-polarizable force fields; $\delta t = 0.2$ fs for the flexible models, which was increased to $\delta t = 0.5$ fs for temperatures $T < 200$ K (for the NEMD simulations, T corresponds to the target temperature); and $\delta t = 2$ fs for the SW potential, which was increased to $\delta t = 5$ fs for $T < 200$ K. 3D periodic boundary conditions were applied in all simulations.

For the empirical force fields (TIP4P/2005, TIP4P/2005f, SPC/E, SPC, TIP3P and TIP5P), a cutoff of 13 Å was used for the oxygen-oxygen Lennard-Jones (LJ) potential, and tail corrections[5] were *not* applied in order for the isotropic equilibrium simulations to be fully consistent with the non-isotropic NEMD simulations. Electrostatic interactions were handled using the P^3M implementation[6] of the Ewald summation method, with a root mean square (RMS) error in per-atom forces of 10^{-5} relative to the force two point charges of elementary charge exert on each other at a distance of 1 Å. For the ReaxFF charge equilibration procedure, a relative residual norm tolerance of 10^{-6} was used for the employed Preconditioned Conjugate Gradient method.[7] The ReaxFF models use an interaction cutoff of 10 Å, which is part of the force field parameterization.

We note that our simulations of TIP4P/2005f in LAMMPS implement a constant O-M distance of 0.1546 Å that does not change with molecular geometry. Thus, small deviations from the canonical TIP4P/2005f model[8] are expected. For the TC, these deviations are expected to be smaller than the uncertainties.

For the SW potentials, we observe freezing in some simulations at the lowest temperatures explored in this work (typically the lowest 1-2 temperatures in Fig. 1 of the main text for each SW model). These MD trajectories are discarded, and our analysis corresponds to the supercooled liquid state for these (P, T) conditions.

1.1 Non-equilibrium molecular dynamics simulations

Boundary-driven non-equilibrium molecular dynamics (NEMD) simulations were performed to calculate the thermal conductivity. In all cases, an elongated (tetragonal) simulation cell of dimensions (L_x, L_y, L_z), where $L_x = L_y = L_\perp$, was used. Two thermostatting regions, set to temperatures T_c (cold) and T_h (hot), of width Δz were located in the centre and edges of the box,

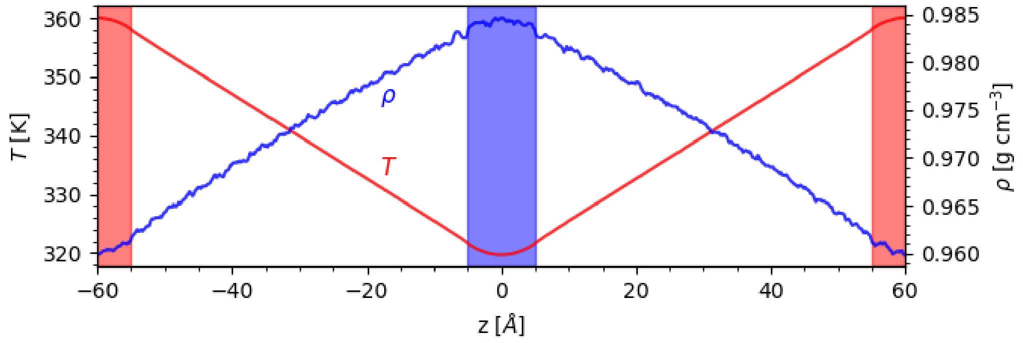

Figure S1: Representative temperature T and density ρ profiles for the NEMD simulations. The blue (cold) and red (hot) indicate the location of the thermostatting regions in the simulation cell. The profiles correspond to TIP4P/2005 and $P = (1 \pm 1)$ bar.

Table 1: NEMD simulation parameters. Symbol meanings are defined in the main SI text.

Force field	N_{mol}	$L_{\parallel}$, L_z [Å]	$T_h - T_c$ [K]	Δz [Å]	Thermostat (τ [fs])	N_{rep}	t_{stat}, t_{prod} [ns]
Rigid[a]	14,400	variable, 120	40	10	CSVR(50)	10	≥1 , 5-20
TIP4P/2005f	14,400	variable, 120	40	10	CSVR(50)	10	≥0.2, 2.4-5.0
TIP5P	14,400	variable, 120	40	10	Langevin(100)	10	≥1, 3-12
ReaxFF	1563	variable, 75	40	5	CSVR(50)	10	≥0.2, 1-4
mW/SW	14,400	variable, 120	10	10	CSVR(50)	10-30	≥2, 20

[a] TIP4P/2005, SPC/E, SPC and TIP3P.

respectively, and extended in the (x, y) plane. Depending on the force field, either the CSVR or Langevin thermostat, with a time constant τ, was used. The system's centre-of-mass velocity was subtracted from each atom at every timestep to ensure linear momentum conservation. For each system, sampling consisted of N_{rep} statistically independent replicas, which were run for a time t_{stat} to establish the stationary state, followed by t_{prod} for data collection. The number of molecules, N_{mol}, in the simulation cell was also varied depending on the force field. The simulation details are summarized in Table 1.

In the stationary state, the heat flux across the system, $\boldsymbol{J}_q = (0, 0, \pm J_q)$, can be obtained from the continuity equation

$$J_q = \frac{|\langle \Delta U \rangle|}{2\delta t A} \tag{1}$$

where $A = L_x \times L_y$ is the cross-sectional area of the simulation box, δt is the timestep, and ΔU is the internal energy exchanged at each timestep, between each thermostatting region and its thermal reservoir. The pressure of the stationary state is given by the average pressure tensor component in the direction of the heat flux, i.e., the z-direction.

The thermal conductivity λ was calculated from the NEMD simulations using Fourier's Law,

$\boldsymbol{J}_q = -\lambda(z)\nabla T(z)$, where $\boldsymbol{J}_q$ is the heat flux and $\nabla T(z)$ is the local temperature gradient. Local densities ρ and temperatures T were determined from a 5 Å bin taken along the z-axis of the simulation cell (the direction parallel to $\boldsymbol{J}_q$). Local temperature gradients were determined by fitting a straight line to the temperature profile within a range of ±12 Å (±8 Å for ReaxFF) around the selected state point.

1.2 Equilibrium simulations

Equilibrium MD simulations were performed in the NPT ensemble. Temperature (pressure) was controlled by the Nosé-Hoover chain thermostat (barostat), with 3 chains, and a time constant of 0.5 ps (2 ps). Cubic simulation cells containing 2133 molecules were used for the rigid non-polarizable, TIP4P/2005f and SW models, while cells containing 521 molecules were used for the ReaxFF models. Each system was first equilibrated for ≥200 ps. Replicas were spawned from this equilibrated configuration, and run for an additional 100 ps in the NVT ensemble using CSVR thermostats (time constant τ = 0.5 ps) with different random seeds to generate statistically independent trajectories. Following this, an additional ≥100 ps of equilibration was performed in the NPT ensemble. Sampling consisted of N_{rep} replicas, each with a production length of t_{prod}. N_{rep} =10-50 and t_{prod} =5 ns for the rigid non-polarizable force fields; N_{rep} =50-100 and t_{prod} =1 ns for TIP4P/2005f; N_{rep} =10-30 and t_{prod} =1-2 ns for the ReaxFF models; and N_{rep} =5-20 and t_{prod} =100 ns for the SW potential.

Thermodynamic properties were calculated from the NPT simulations as follows. The thermal expansion coefficient α_P, isothermal compressibility β_T and isobaric heat capacity C_P were obtained from the fluctuation relations:

$$\alpha_P = \frac{\langle \delta V \delta H \rangle_{NPT}}{k_{\mathrm{B}} T^2 V}\,, \quad \beta_T = \frac{\langle \delta V^2 \rangle_{NPT}}{V k_{\mathrm{B}} T}\,, \quad C_P = \frac{\langle \delta H^2 \rangle_{NPT}}{k_{\mathrm{B}} T^2} \tag{2}$$

where T is the temperature, P is the pressure, V is the volume, H is the enthalpy, and k_{B} is the Boltzmann constant. $\delta\mathcal{A} = \mathcal{A} - \langle\mathcal{A}\rangle_{\mathrm{ens}}$ and $\langle\mathcal{A}\rangle_{\mathrm{ens}}$ is the ensemble average of variable $\mathcal{A}$. The isentropic compressibility β_S and isentropic speed of sound c_S can then be calculated from

$$\beta_S = \beta_T - \frac{\alpha_P^2 T}{\rho C_P}\,, \quad c_S^2 = \frac{1}{\rho \beta_S} \tag{3}$$

where ρ is the density.

The exception to this is for TIP5P, for which thermodynamic response functions were calculated directly from the equation of state using the definitions:

$$\alpha_P = -\frac{1}{V}\left(\frac{\partial V}{\partial T}\right)_P\,, \quad \beta_T = -\frac{1}{V}\left(\frac{\partial V}{\partial P}\right)_T\,, \quad C_P = \left(\frac{\partial H}{\partial T}\right)_P \tag{4}$$

2 Additional results, data and discussion

2.1 Additional discussion: the Bridgman equation in supercooled water

More generally than the TCM, the Bridgman equation has been used to predict the anomalous behaviour of liquid water. Molecular simulations predict the existence of a thermal conductivity *minimum* in supercooled water at temperatures below those currently accessible to experiments.[9–11] Recent non-equilibrium simulations of TIP4P/2005 (one of the most accurate general purpose water model for thermodynamic properties[12]) place the TC minimum at ∼220 K at near-standard pressures.[11] In good agreement, the Bridgman equation for TIP4P/2005 predicts a temperature of ∼230 K.[10] Furthermore, the Bridgman equation accurately reproduces the experimental TC of supercooled water down to 273.15 K at 1 bar, which is the limit of validity of the experimental IAPWS-2011 correlation[13]. Extrapolating to lower temperatures, the Bridgman equation predicts a TC minimum at ∼230 K.[14] Mode-coupling theory suggests that critical fluctuations associated with the hypothesized liquid-liquid critical point would have a negligible effect on the TC minimum and "the behavior of [supercooled water's] thermal conductivity can be fully explained by the anomalies of the thermodynamic properties".[14] Similarly, at near-standard pressures the liquid-gas critical point has a negligible effect on the TC of liquid water (and therefore the TCM) with a critical enhancement $\lesssim 0.1$ % for $T < 450$ K, which is smaller than the uncertainties associated with the TC values.[13]

2.2 Numerical data for T_{ex}

Table 2: The temperature T_{ex} at which extrema in thermophysical properties occur as a function of temperature at 10 bar, as predicted by selected force fields. The temperatures of the density maximum $T_{\max(\rho)}$, isothermal compressibility minimum $T_{\min(\beta_T)}$, isentropic compressibility minimum $T_{\min(\beta_S)}$, isentropic speed of sound maximum $T_{\max(c_S)}$, Bridgman thermal conductivity maximum $T_{\max(\lambda_{\text{B}})}$, thermal conductivity maximum $T_{\max(\lambda)}$, and the quantum-corrected thermal conductivity maximum $T_{\max(\lambda^{\text{qc}})}$.

Force Field	$T_{\max(\rho)}$ [K]	$T_{\min(\beta_T)}$ [K]	$T_{\min(\beta_S)}$ [K]	$T_{\max(c_S)}$ [K]	$T_{\max(\lambda_{\text{B}})}$ [K]	$T_{\max(\lambda)}$ [K]	$T_{\max(\lambda^{\text{qc}})}$ [K]
TIP4P/2005	277(1)	309(3)	326(4)	333(5)	321(3)	335(5)	370(9)
TIP4P/2005f	271(1)	300(4)	312(4)	320(5)	310(4)	322(8)	360(18)
SPC/E	248(1)	262(3)	272(4)	280(6)	270(4)	291(5)	344(12)
SPC	224(1)	251(4)	262(4)	271(5)	260(3)	266(7)	314(12)
TIP3P	199(1)	235(2)	249(4)	258(4)	246(3)	246(4)	294(12)
TIP5P	282.3(6)	None	None	None	None	304(7)	319(15)
water-2017	205(6)	None	None	None	None	None	None
CHON-2017_weak	254(3)	None	None	None	None	None	313(21)
mW	251(1)	298(1)	322(2)	332(2)	320(2)	246(4)	N/A
Exp.[a]	277	320	337	347	334	404	N/A

[a] Experimental data are from, or calculated from, Refs.[13,15].

2.3 Thermodynamic response functions

For the SW potentials, we note that at $\phi = 17, 18, 27$ the thermal expansion coefficient α_P features a minimum with $\min(\alpha_P) > 0$. For $\phi = 19$ extrapolating α_P to $T = 0$ gives $\alpha_P > 0$ for the entire temperature range. This implies that the density maximum is not observed at lower temperatures at these values of ϕ.

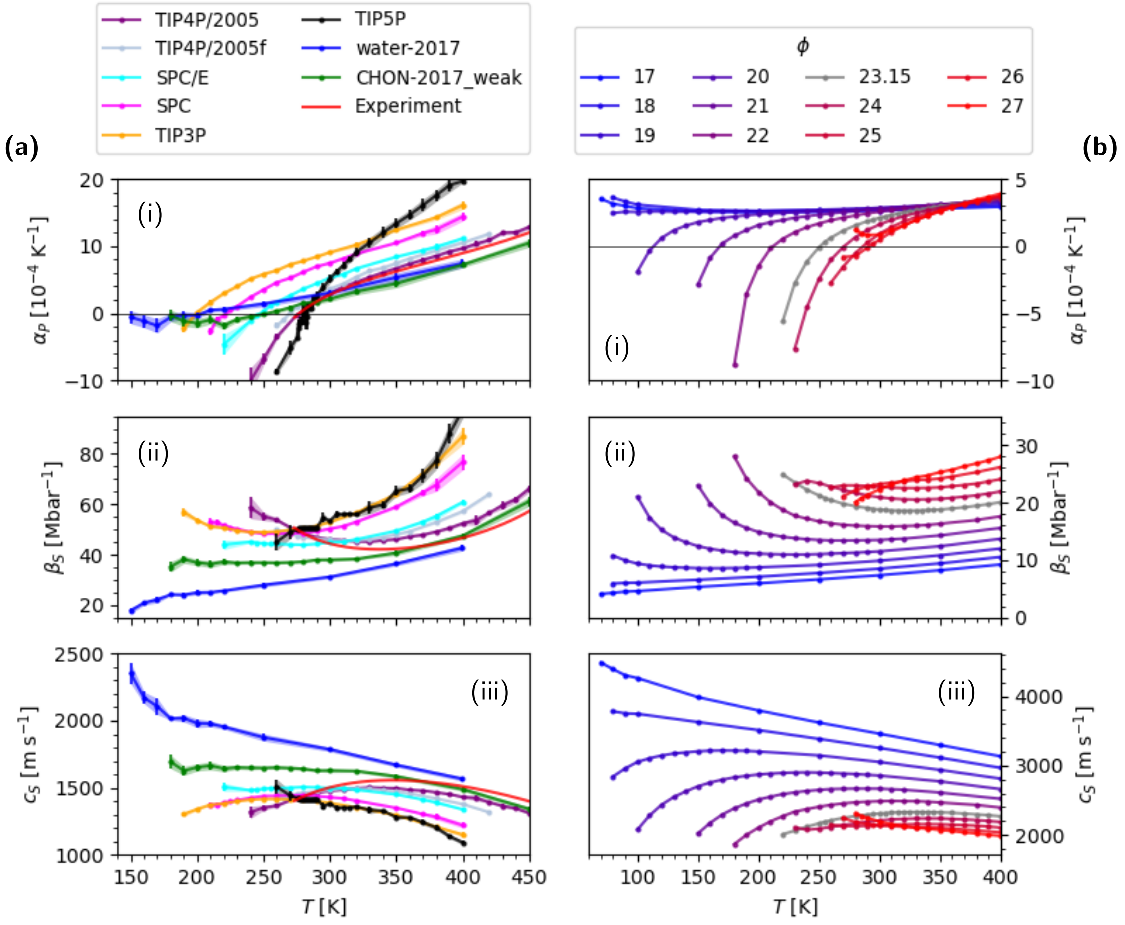

Figure S2: Thermodynamic response functions of (a) atomistic force fields of water and (b) SW potentials as a function of temperature T at constant pressure: the (i) thermal expansion coefficient α_P, (ii) isentropic compressibility β_S, and (iii) isentropic speed of sound c_S. Data corresponds to 10 bar.

2.4 Density maximum of TIP5P

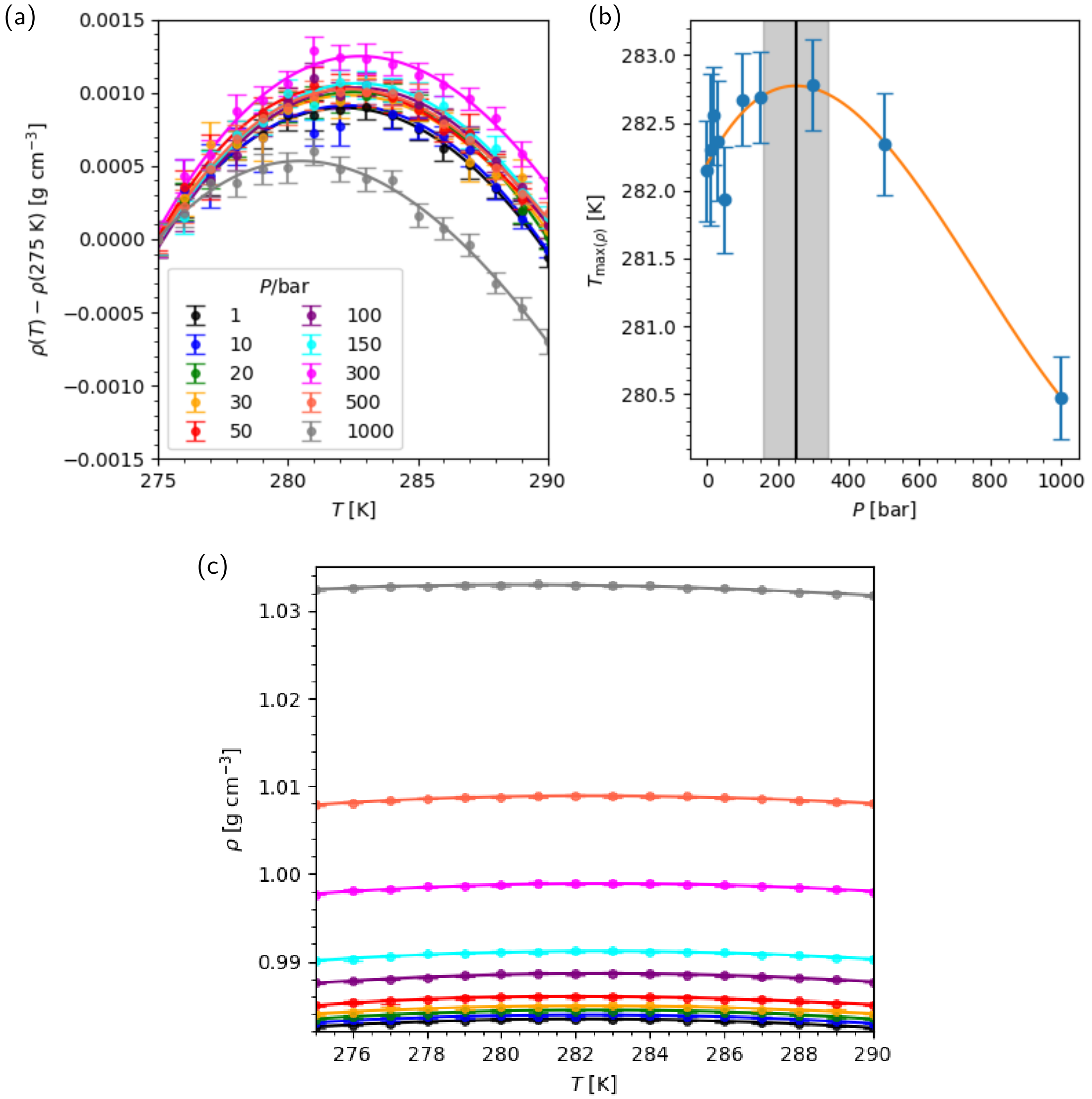

Figure S3: The density maximum of TIP5P at different pressures P. (a) The density $\rho(T)$ as a function of temperature T and shifted by $\rho(T = 275\text{ K})$ for clarity. (b) The temperature of maximum density $T_{\max(\rho)}$ as a function of pressure. The solid black vertical line denotes the pressure of the maximum $T_{\max(\rho)}$ and the grey shaded region denotes its associated uncertainty. (c) ρ as a function of T. In all cases, solid lines are cubic functions fit to the data.

2.5 Quantum correction to the heat capacity

In this work we employ the method[16] of Berens to quantum-correct C_P within a harmonic oscillator approximation.[16]

$$C_P^{\mathrm{qc}} = C_P + C^{\Delta} \tag{5}$$

This involves integrating $S(\nu)$ with a weighting function W_C^{Δ}, which is the difference between the quantum and classical weighting functions for a set of harmonic oscillators:

$$C^{\mathrm{c}} = k_{\mathrm{B}} \int_0^{\infty} S(\nu) W_C^{\mathrm{c}} d\nu, \quad W_C^{\mathrm{c}} = 1 \tag{6}$$

$$C^{\mathrm{q}} = k_{\mathrm{B}} \int_0^{\infty} S(\nu) W_C^{\mathrm{q}} d\nu, \quad W_C^{\mathrm{q}} = \frac{u^2 e^u}{(1-e^u)^2} \tag{7}$$

$$C^{\Delta} = C^{\mathrm{q}} - C^{\mathrm{c}} = k_{\mathrm{B}} \int_0^{\infty} S(\nu) W_C^{\Delta} d\nu, \quad W_C^{\Delta} = \frac{u^2 e^u}{(1-e^u)^2} - 1 \tag{8}$$

where $u = h\nu/(k_{\mathrm{B}}T)$ and h is Planck's constant. C^i and W_C^i are the heat capacities and corresponding weighing functions for the sets of classical ($i = \mathrm{c}$) and quantum ($i = \mathrm{q}$) harmonic oscillators. $S(\nu)$ was obtained from the Fourier transform of the total VACF $C(t)$, defined as the mass-weighted sum of atomic VACFs,

$$C(t) = \sum_{j=1}^{N} m_j \langle \boldsymbol{v}_j(t) \cdot \boldsymbol{v}_j(0) \rangle \tag{9}$$

$$S(\nu) = \frac{2}{k_{\mathrm{B}}T} \lim_{t' \to \infty} \int_{-t'}^{t'} C(t) e^{-i2\pi\nu t} dt \tag{10}$$

where m_j and $\boldsymbol{v}_j$ are the mass and velocity of atom j, respectively, and t is the elapsed time from an arbitrary starting time. $S(\nu)$ has the normalization $\int_0^{\infty} S(\nu) dv = N$ where N is the total number of degrees of freedom.

This approach satisfies the correspondence principle $\lim_{\hbar \to 0} C_V^{\mathrm{qc}} = C_V$. While C_V^{Δ} does not incorporate anharmonic effects, they are included through the classical C_V. The quantum correction C_V^{Δ} is based on the division of dynamics in frequency space. The low-frequency region features major anharmonic effects but behaves nearly classically, so while the correction is less accurate, it is small. At higher frequencies, atomic motions are better approximated as harmonic and quantum effects are more significant, so while the correction is large, it is also reasonably accurate. Thus, the quantum correction can be applied over the entire frequency domain.

C^{Δ} values were calculated from MD simulations in the NVT ensemble at the density corresponding to $P = 10$ bar. For the flexible models, we partition C^{Δ} into intramolecular and intermolecular contributions by dividing $S(\nu)$ at frequency ν_{cut} corresponding to the minimum between the libration peak and bending peak of a smoothed $S(\nu)$. The value of ν_{cut} therefore depends on the force field and thermodynamic conditions, and falls in the range 1200-1600 cm^{-1} for the thermodynamic states considered in this work.

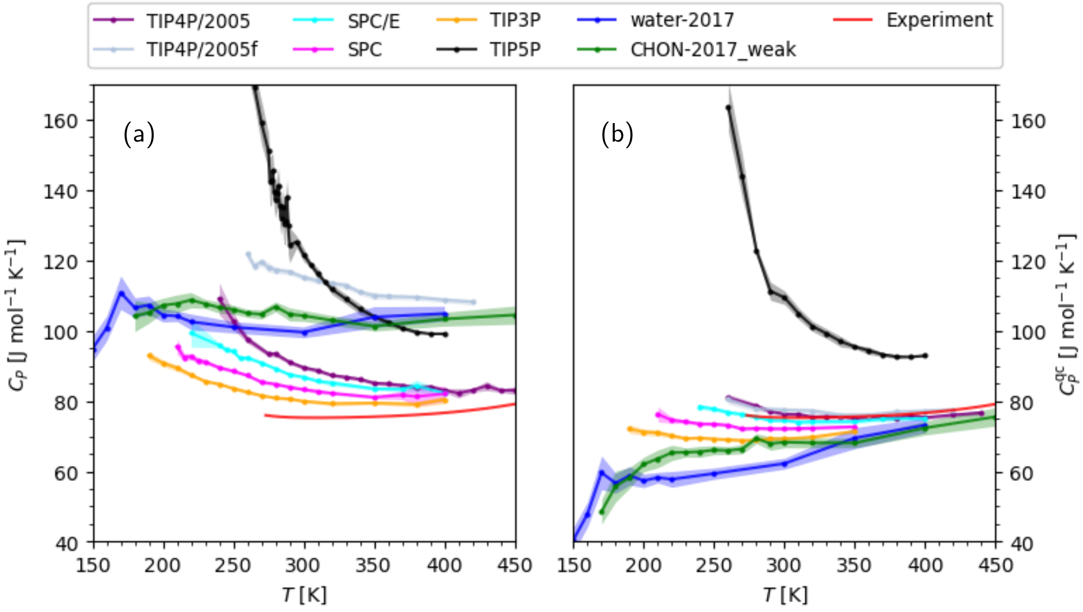

Figure S4: (a) Isobaric heat capacity C_P and (b) quantum-corrected isobaric heat capacity C_P^{qc} for selected atomistic force fields. Data corresponds to 10 bar.

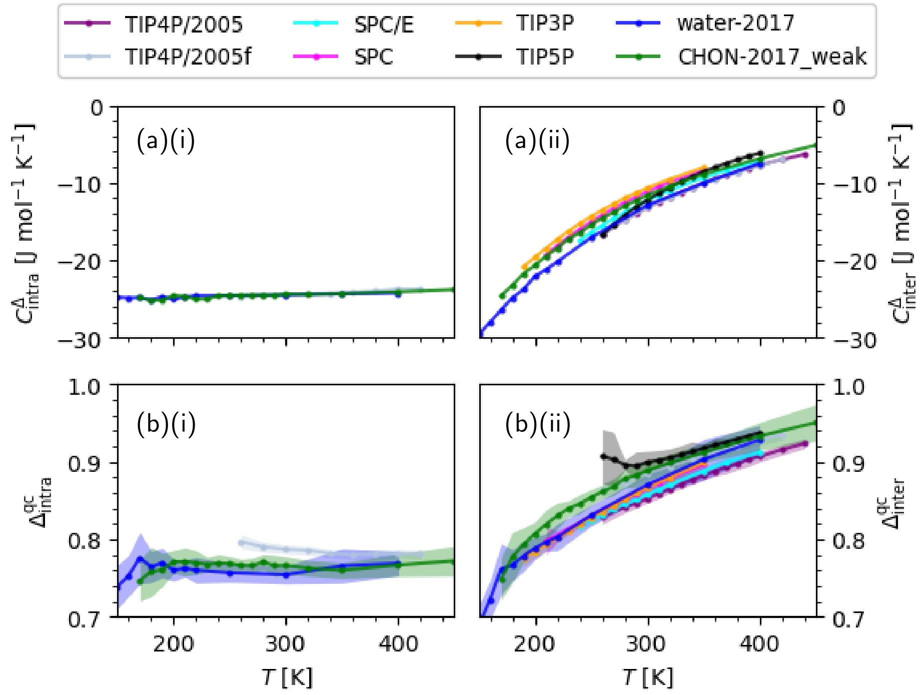

Figure S5: Intra- (i) and intermolecular (ii) contributions to the quantum corrections for selected atomistic force fields. (a) The quantum-correction to the heat capacity C^{Δ} and (b) Δ^{qc} (see the main text). Data corresponds to 10 bar.

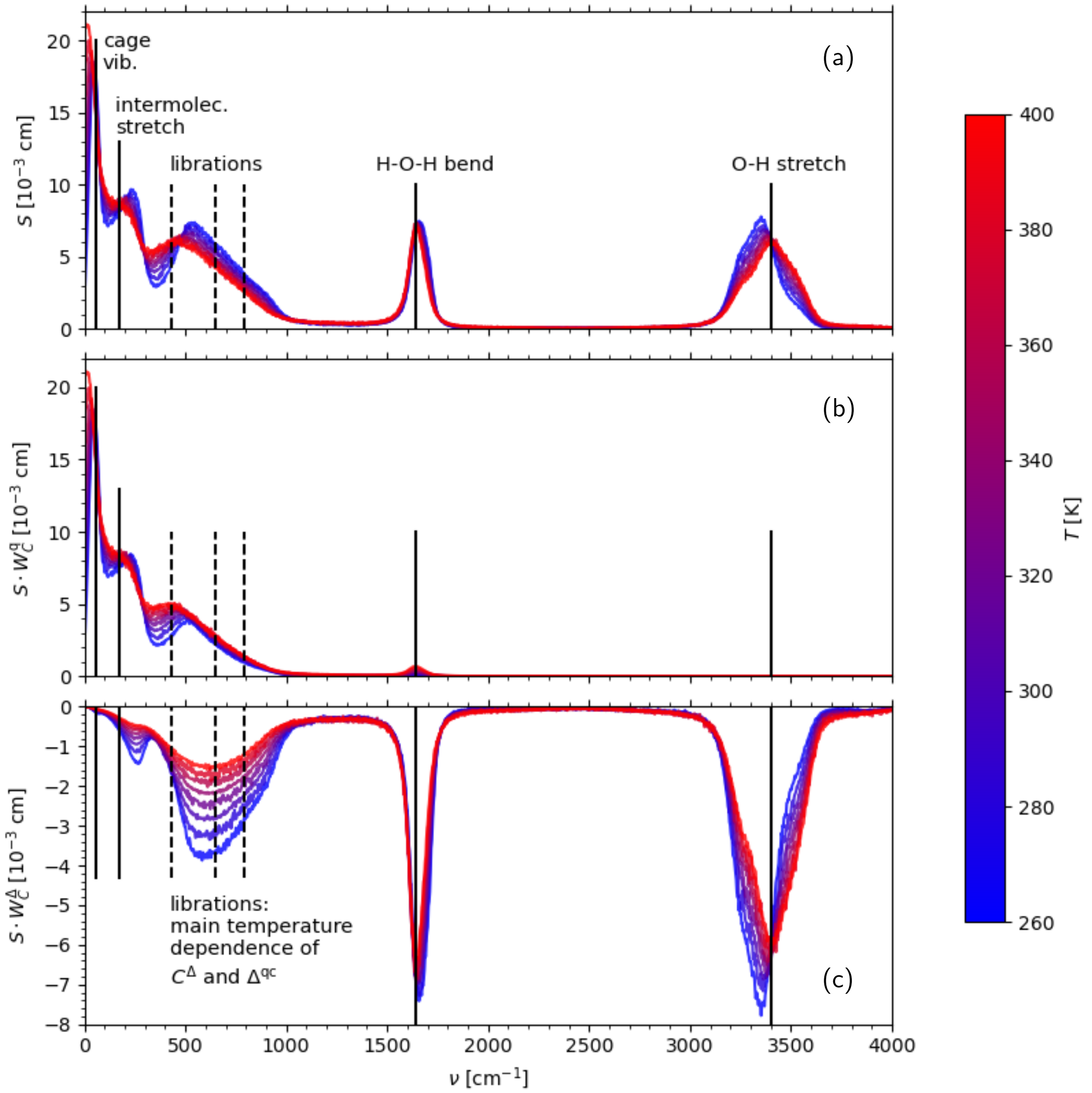

Figure S6: Classical and quantum mass-weighted velocity spectra of TIP4P/2005f at 10 bar: (a) the classical $S(\nu)$, (b) the quantum $S(\nu)W_C^{\mathrm{q}}$ and (c) their difference $S(\nu)W_C^{\Delta}$. The spectra correspond to temperatures T/K = 260, 280, 300, 320, 340, 360, 380 and 400. Experimental lines are from Refs.[17–20].

2.6 Orientational tetrahedral order parameter for the SW potentials

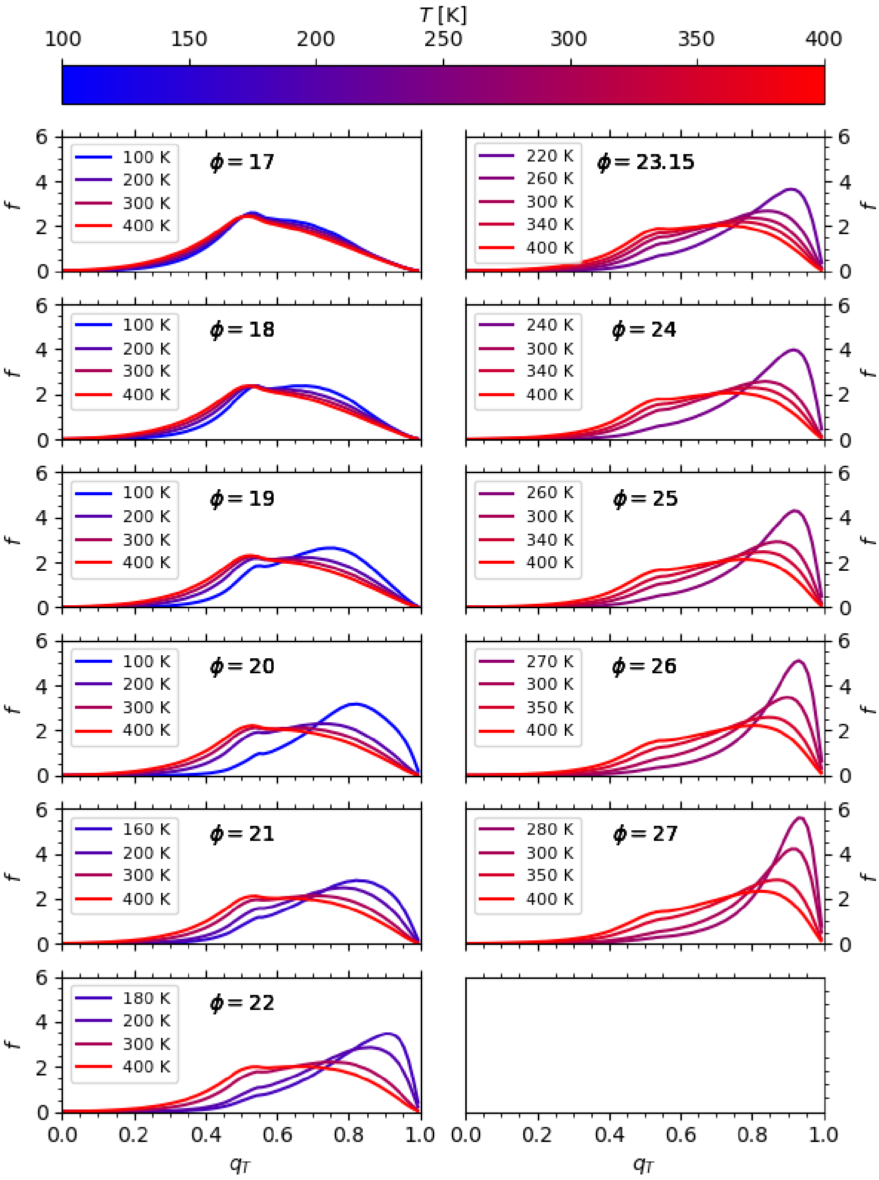

Figure S7: Probability density functions f of the orientational tetrahedral order parameter q_T for the SW potentials $17 \leq \phi \leq 27$ at different temperatures T.

2.7 Thermal conductivity

2.7.1 Interpolating to P =10 bar

The thermal conductivity at 10 bar was interpolated by fitting a straight line to the $\lambda(P;T)$ data at a given T. Between 2-5 data points at pressures close to 10 bar were used for each temperature. For a given T, NEMD simulations corresponding to different pressures were achieved by varying the density (via $L_\perp$) by $\sim 10^{-3}$ g cm^{-3} for the atomistic force fields, and by $\sim 10^{-4}$ g cm^{-3} for the SW potentials. Additionally, the reduced computational cost of the SW potential facilitates the use of small temperature differences $T_h - T_c = 10$ K, corresponding to $\nabla T < 2$ K nm^{-1}, and consequently allows us to target very accurately specific thermodynamic states.

(a)

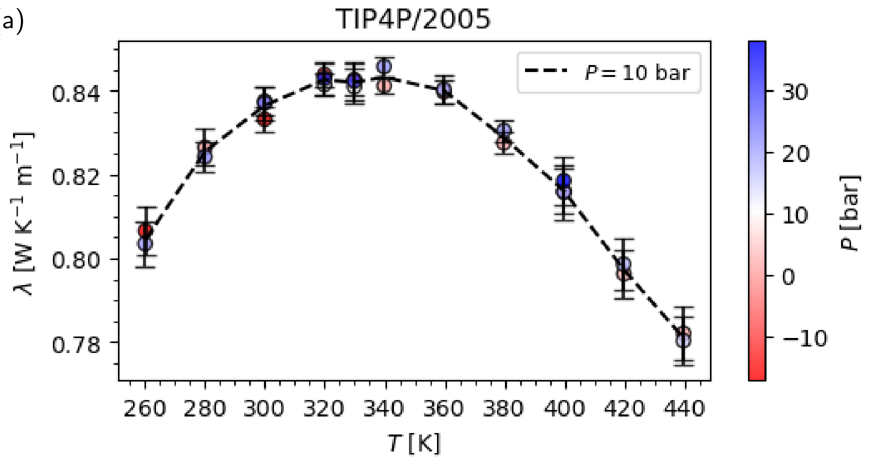

(b)

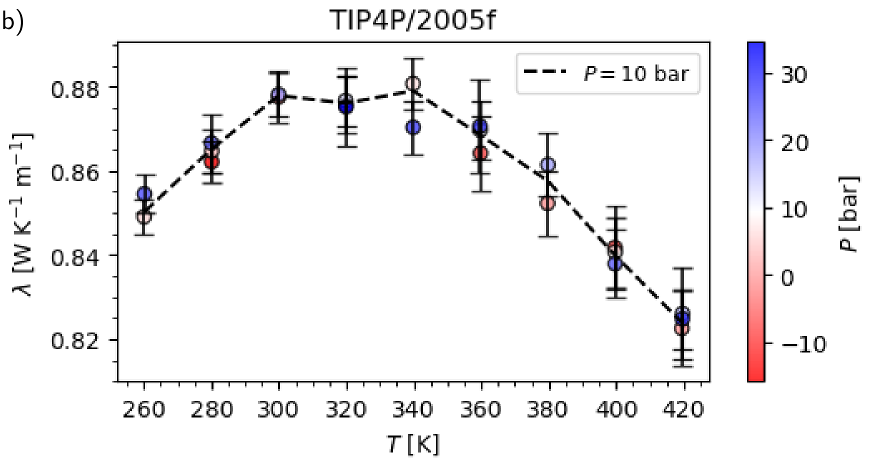

(c)

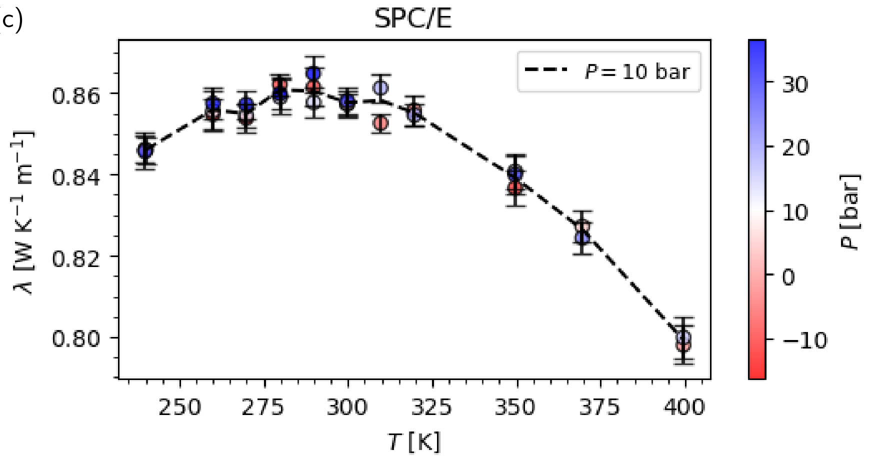

SPC/E
P = 10 bar
λ [W K⁻¹ m⁻¹]
T [K]
P [bar]
0.80
0.82
0.84
0.86
250
275
300
325
350
375
400
−10
0
10
20
30

(d)

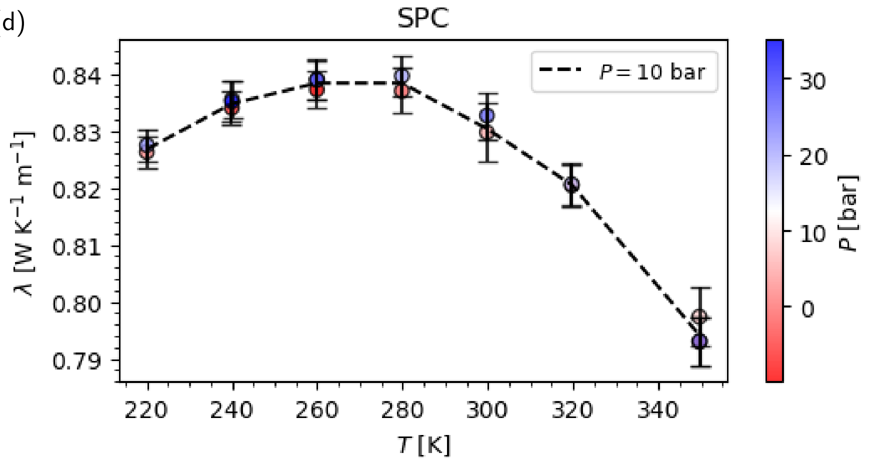

SPC
P = 10 bar
λ [W K⁻¹ m⁻¹]
T [K]
P [bar]
0.79
0.80
0.81
0.82
0.83
0.84
220
240
260
280
300
320
340
0
10
20
30

(e)

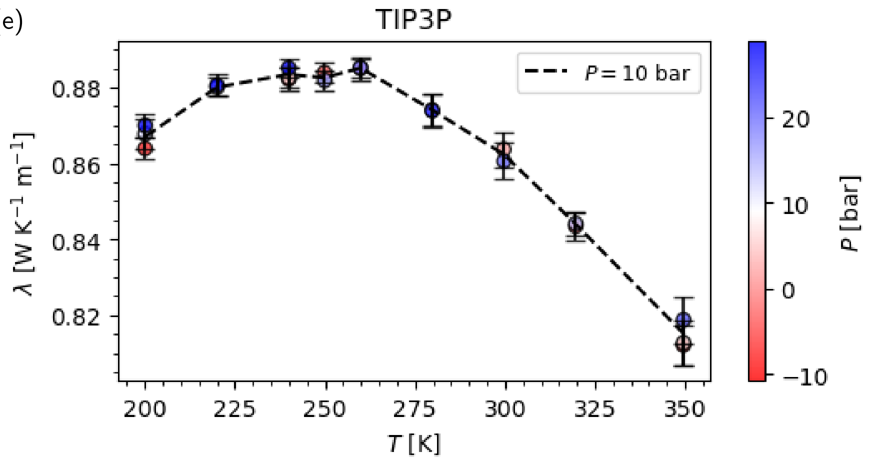

TIP3P
P = 10 bar
λ [W K⁻¹ m⁻¹]
T [K]
P [bar]
0.82
0.84
0.86
0.88
200
225
250
275
300
325
350
−10
0
10
20

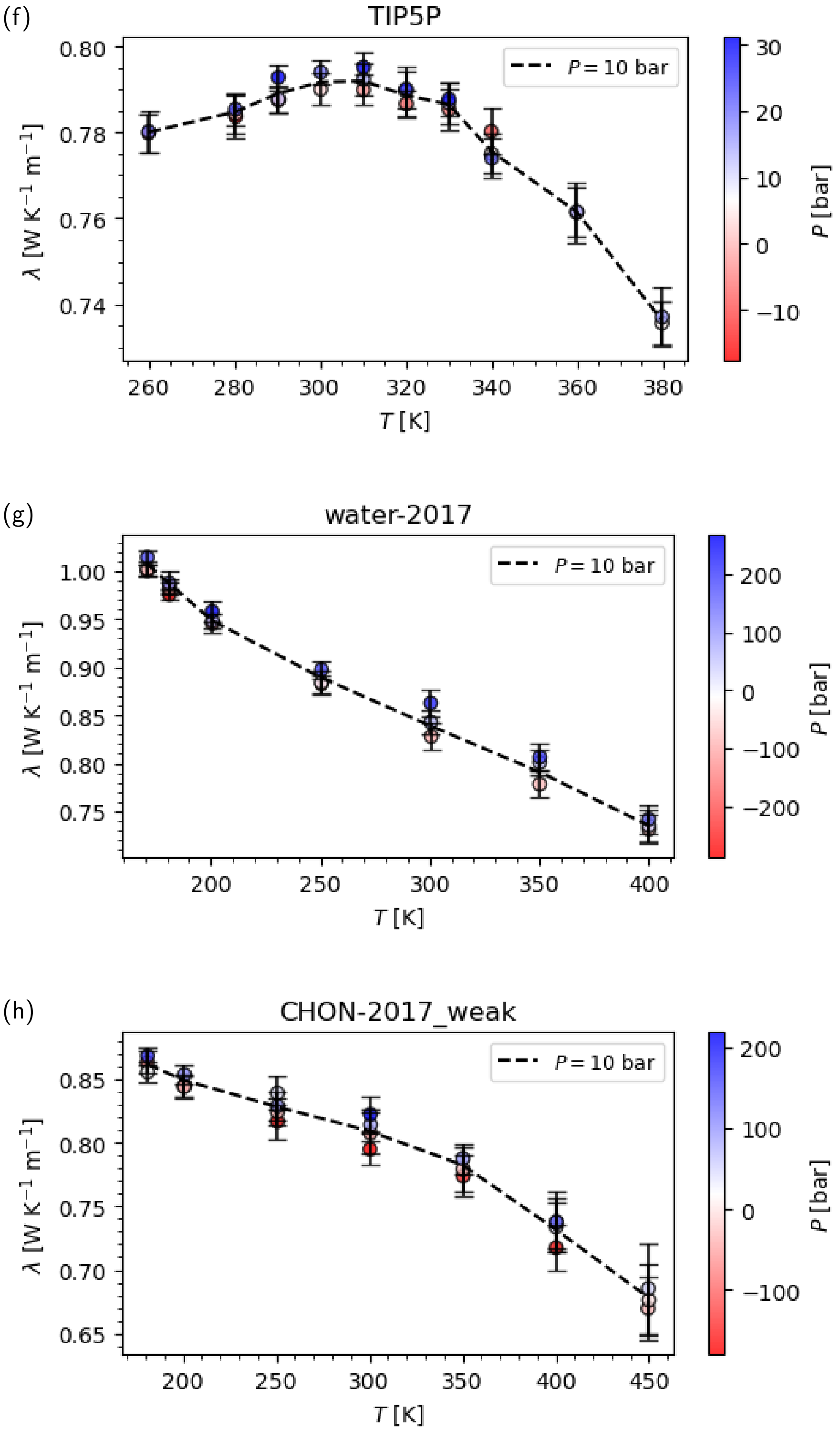

Figure S8: Thermal conductivity λ as a function of temperature T for selected atomistic force fields. (a) TIP4P/2005, (b) TIP4P/2005f, (c) SPC/E, (d) SPC, (e) TIP3P, (f) TIP5P, (g) water-2017 and (h) CHON-2017_weak. The symbols are colour coded according to pressure P and the dashed lines show the interpolated λ values corresponding to 10 bar.

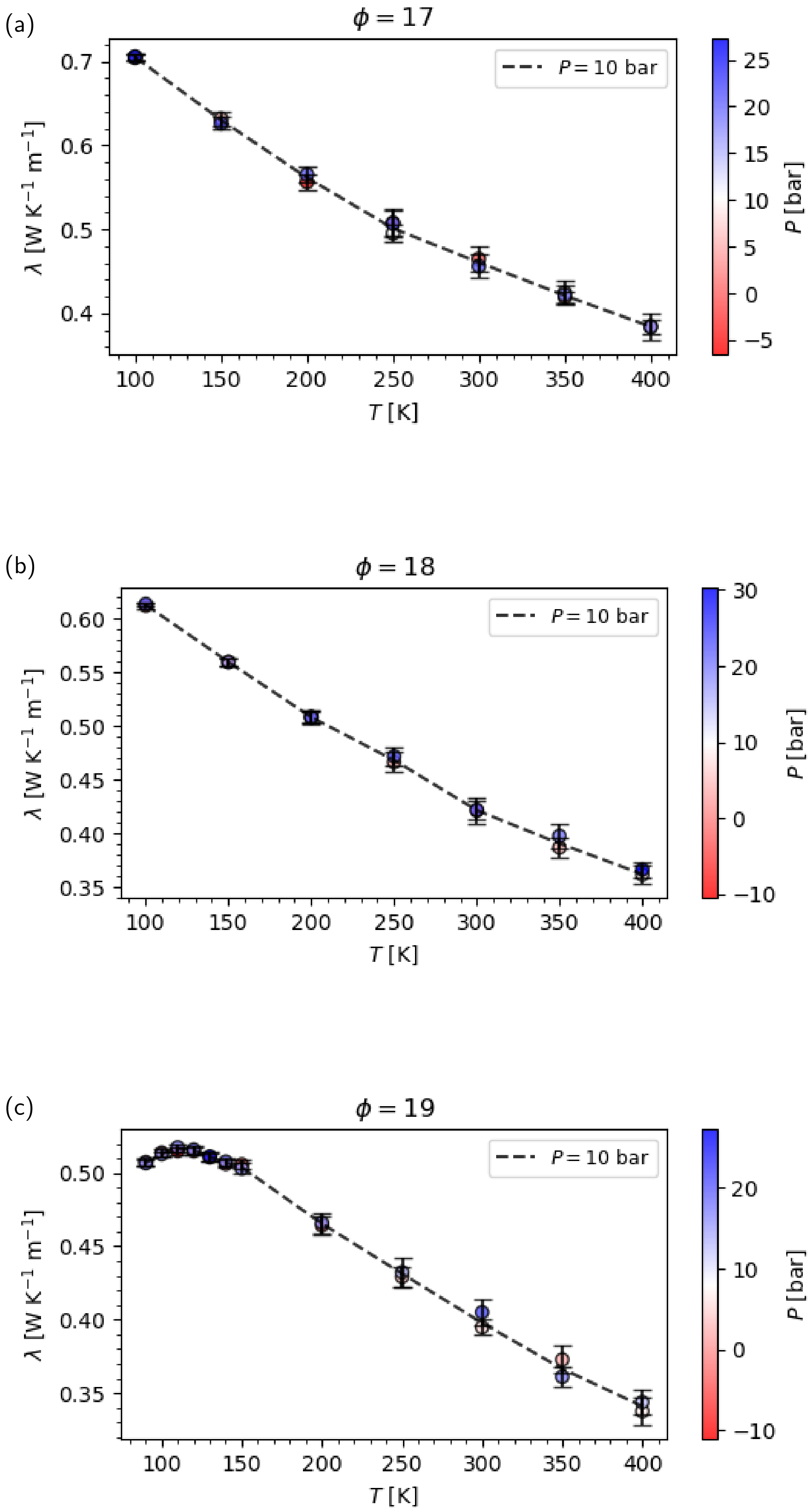
(a)
ϕ = 17
P = 10 bar
λ [W K⁻¹ m⁻¹]
T [K]
P [bar]
(b)
ϕ = 18
P = 10 bar
λ [W K⁻¹ m⁻¹]
T [K]
P [bar]
(c)
ϕ = 19
P = 10 bar
λ [W K⁻¹ m⁻¹]
T [K]
P [bar]

(d)

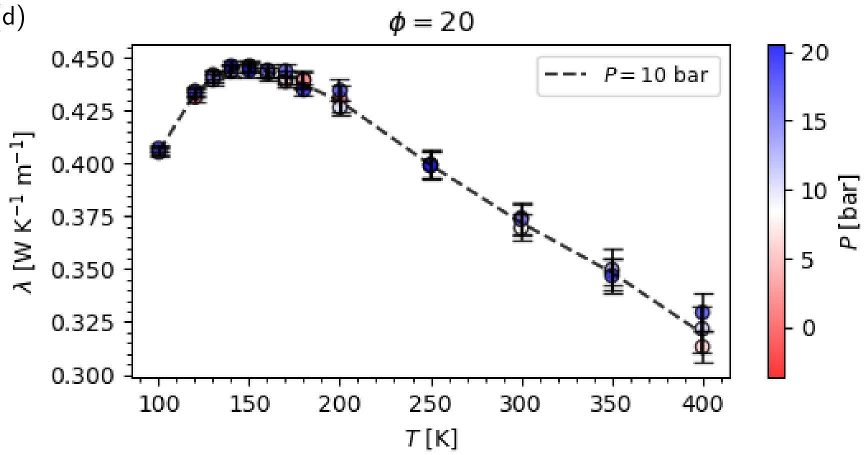

$\phi = 20$
$P = 10$ bar
λ [W K^{-1} m^{-1}]
0.450
0.425
0.400
0.375
0.350
0.325
0.300
100
150
200
250
300
350
400
T [K]
20
15
10
5
0
P [bar]

(e)

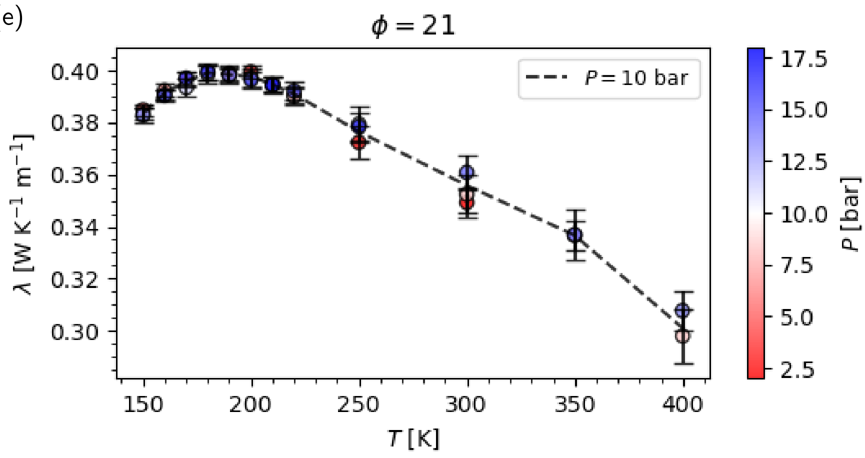

$\phi = 21$
$P = 10$ bar
λ [W K^{-1} m^{-1}]
0.40
0.38
0.36
0.34
0.32
0.30
150
200
250
300
350
400
T [K]
17.5
15.0
12.5
10.0
7.5
5.0
2.5
P [bar]

(f)

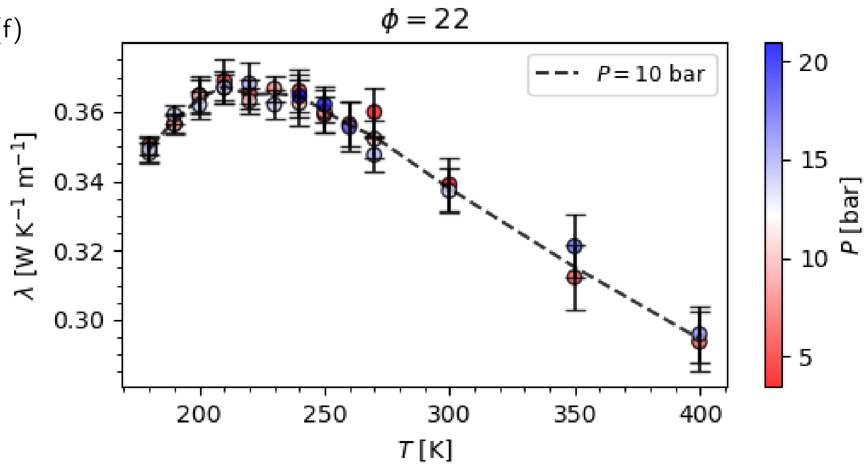

$\phi = 22$
$P = 10$ bar
λ [W K^{-1} m^{-1}]
0.36
0.34
0.32
0.30
200
250
300
350
400
T [K]
20
15
10
5
P [bar]

(g)

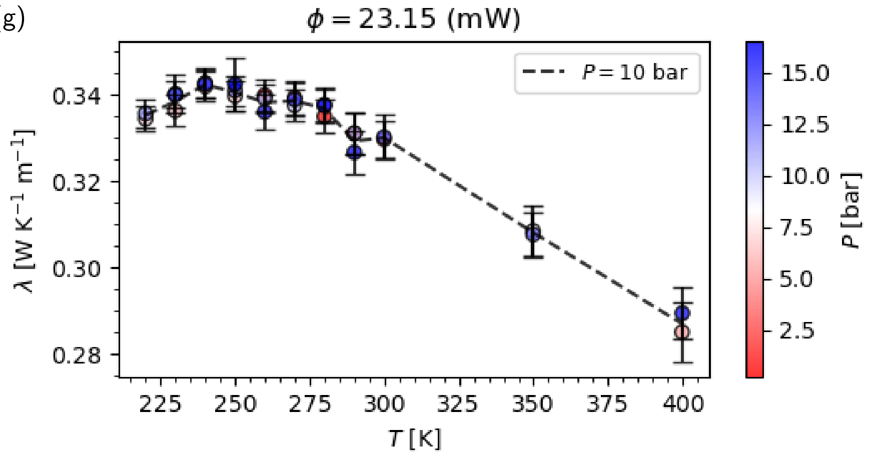
$\phi = 23.15$ (mW)
$P = 10$ bar
0.34
0.32
0.30
0.28
λ [W K^{-1} m^{-1}]
225
250
275
300
325
350
375
400
T [K]
15.0
12.5
10.0
7.5
5.0
2.5
P [bar]

(h)

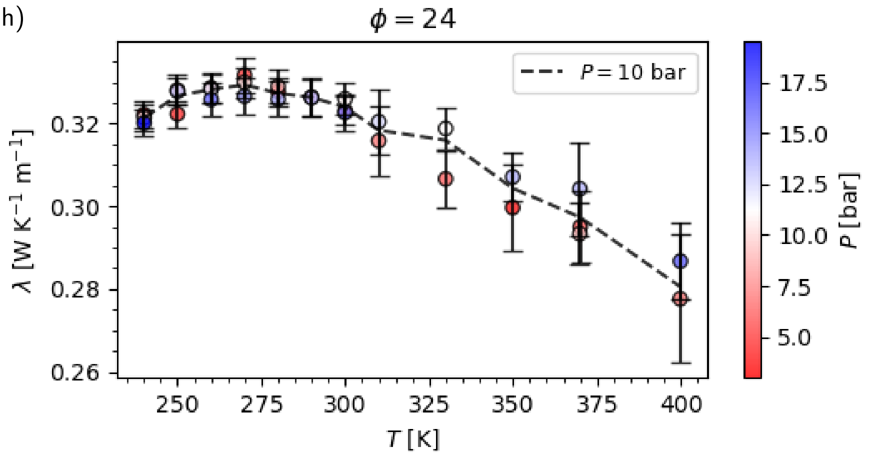
$\phi = 24$
$P = 10$ bar
0.32
0.30
0.28
0.26
λ [W K^{-1} m^{-1}]
250
275
300
325
350
375
400
T [K]
17.5
15.0
12.5
10.0
7.5
5.0
P [bar]

(i)

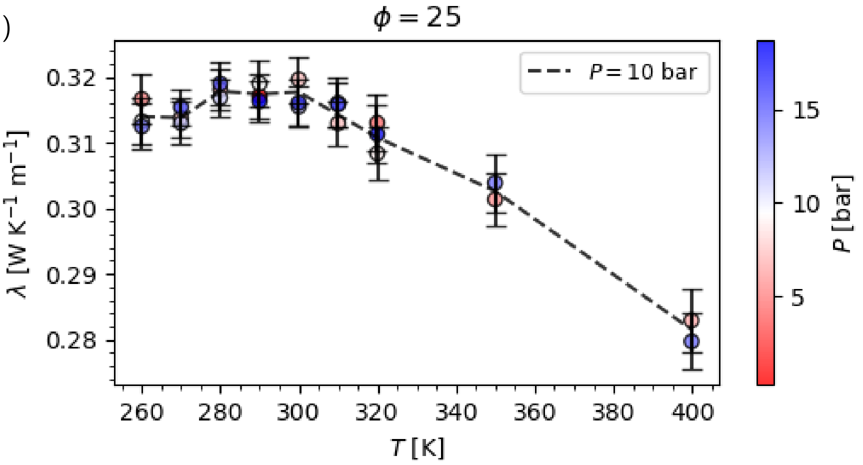
$\phi = 25$
$P = 10$ bar
0.32
0.31
0.30
0.29
0.28
λ [W K^{-1} m^{-1}]
260
280
300
320
340
360
380
400
T [K]
15
10
5
P [bar]

(j)

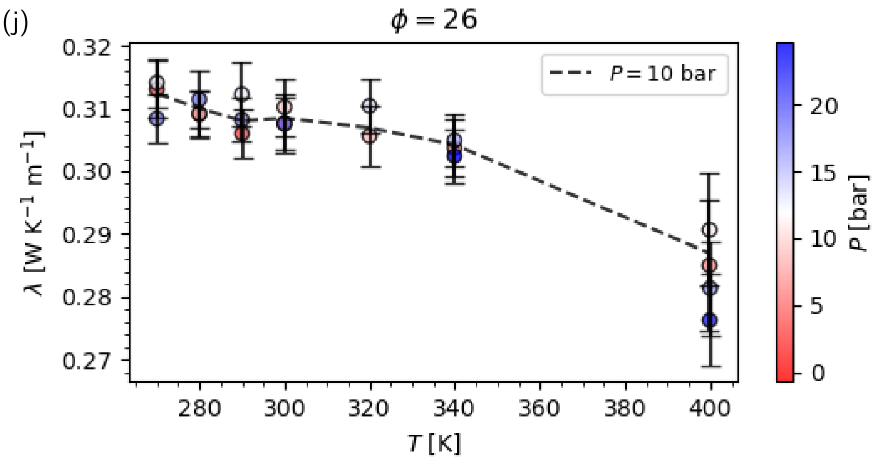

(k)

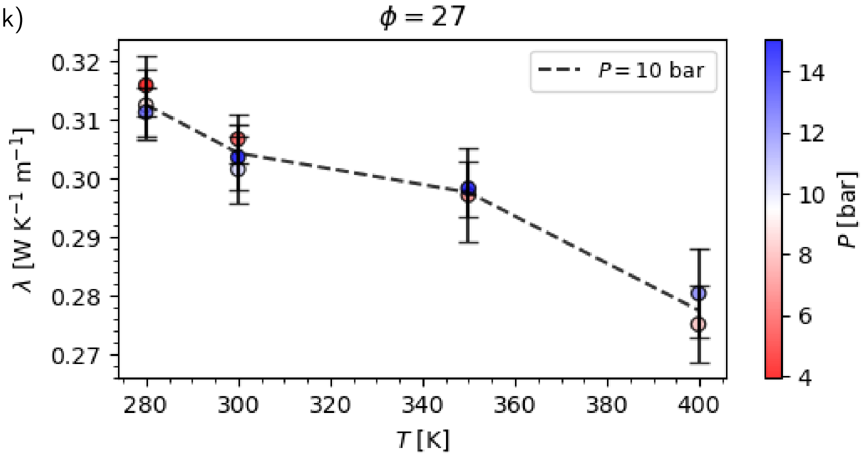

Figure S9: Thermal conductivity λ as a function of temperature T for the SW potentials. The symbols are colour coded according to pressure P and the dashed lines show the interpolated λ values corresponding to 10 bar.

2.7.2 Finite-size effects and convergence

Finite-size effects in TC have rarely[21,22] been investigated in simulations of liquid water because thermal transport in molecular liquids is dominated by nearest-neighbour collisions, setting the characteristic length scale for heat transport at around 1 molecular diameter, and significant finite-size effects are therefore not expected. However, we require very precise TC values to resolve the TCM with differences $\sim 10^{-3}$ W m^{-1} K^{-1} being potentially relevant in this work. This level of precision is smaller or approximately equal to the uncertainties typically reported in simulation studies.[11,21–38]

In order to assess the impact of finite-size effects, we vary the NEMD simulation cell size. As stated in section 1.1 an elongated (tetragonal) simulation cell of dimensions (L_x, L_y, L_z), where $L_x = L_y = L_\perp$, was used. We change L_z and scale $L_\perp$ and the number of molecules N accordingly such that $L_\perp \approx 0.5L_z$ at $\rho \approx 1.0$ g cm^{-3}. For each (T, N, L_z) system, we calculate the TC at P =10 bar by interpolation, as described in section 2.7.1. The temperature difference between the thermostats is set to $T_h - T_c = 40$ K for the empirical force fields, except for $L_z = 60$ Å where it is decreased to $T_h - T_c = 30$ K. For the SW potentials, $T_h - T_c = 10$ K. ∇T therefore decreases as L_z is increased past 80 Å; these large simulation sizes require additional sampling to compensate for the lower signal-to-noise ratio.

We find that $L_z = 120$ Å is sufficient to converge the TC for the empirical and SW models. The increased computational cost of the ReaxFF force fields prohibits system sizes significantly larger than the $L_z = 75$ Å already used. However, these models feature a monotonically increasing $\lambda(T)$, and finite-size effects are therefore not expected to change their phenomenology (i.e., they will not induce the TCM).

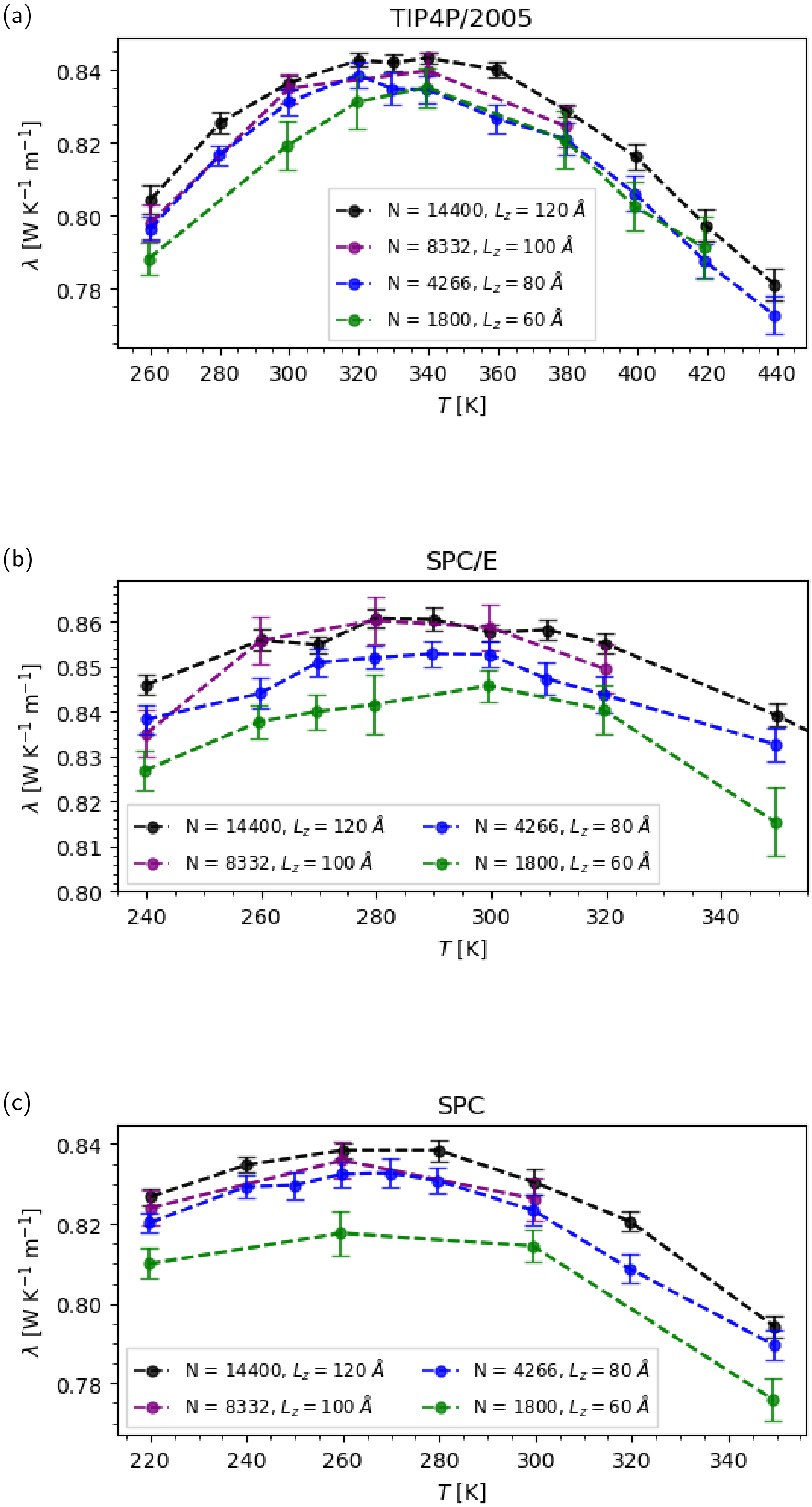
(a)
TIP4P/2005
0.84
0.82
0.80
0.78
λ [W K^{-1} m^{-1}]
N = 14400, $L_z = 120$ Å
N = 8332, $L_z = 100$ Å
N = 4266, $L_z = 80$ Å
N = 1800, $L_z = 60$ Å
260 280 300 320 340 360 380 400 420 440
T [K]
(b)
SPC/E
0.86
0.85
0.84
0.83
0.82
0.81
0.80
λ [W K^{-1} m^{-1}]
N = 14400, $L_z = 120$ Å
N = 4266, $L_z = 80$ Å
N = 8332, $L_z = 100$ Å
N = 1800, $L_z = 60$ Å
240 260 280 300 320 340
T [K]
(c)
SPC
0.84
0.82
0.80
0.78
λ [W K^{-1} m^{-1}]
N = 14400, $L_z = 120$ Å
N = 4266, $L_z = 80$ Å
N = 8332, $L_z = 100$ Å
N = 1800, $L_z = 60$ Å
220 240 260 280 300 320 340
T [K]

(d)

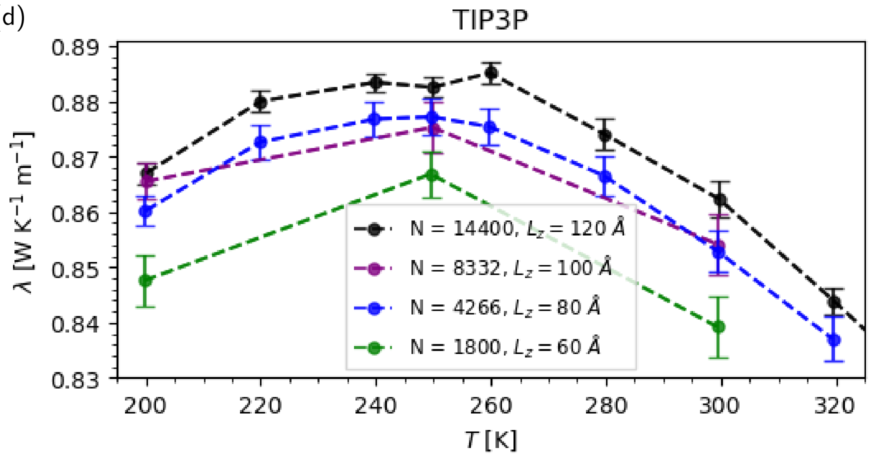

(e)

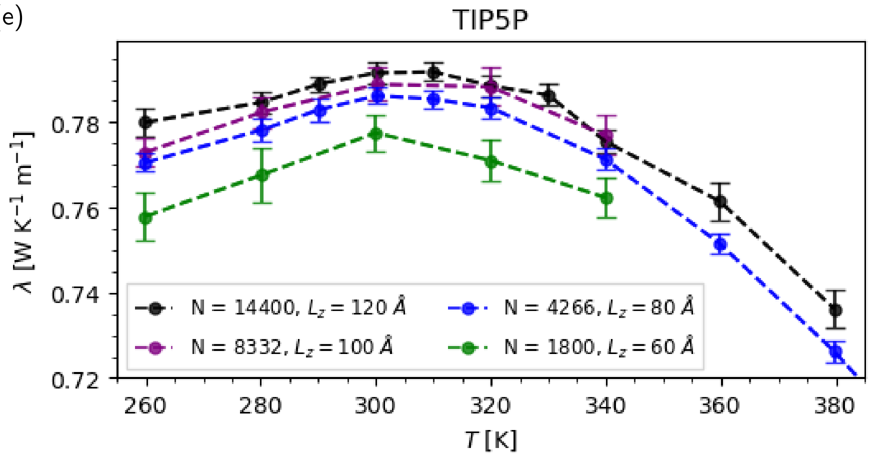

Figure S10: Finite-size convergence of thermal conductivity λ as a function of temperature T for selected atomistic force fields. System size: N is the number of molecules and L_z is the length of the simulation cell in the direction perpendicular to the heat flux. Data corresponds to 10 bar.

(a)

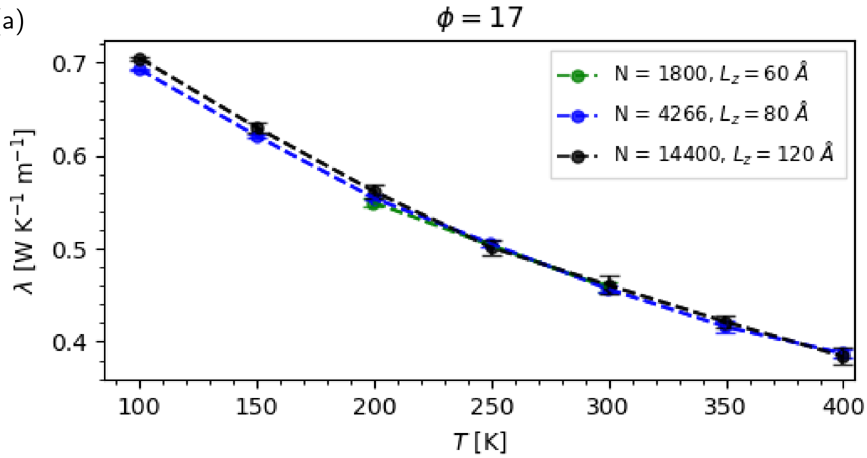

(b)

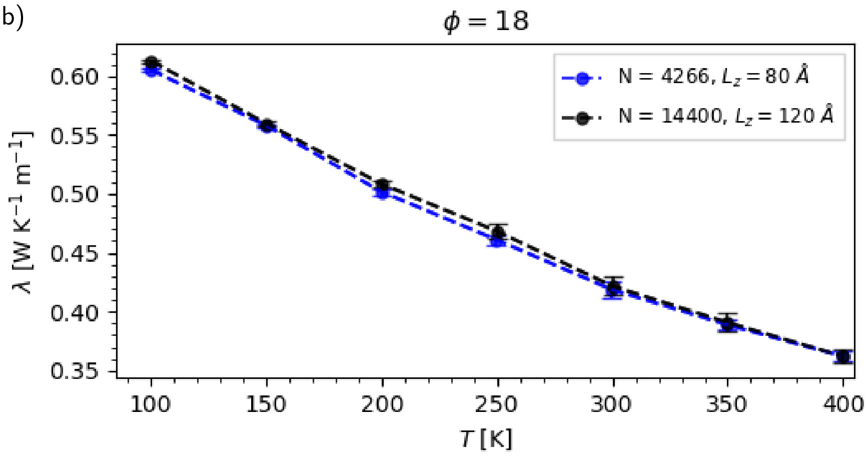

$\phi = 18$
N = 4266, $L_z = 80$ Å
N = 14400, $L_z = 120$ Å
λ [W K^{-1} m^{-1}]
0.60
0.55
0.50
0.45
0.40
0.35
100
150
200
250
300
350
400
T [K]

(c)

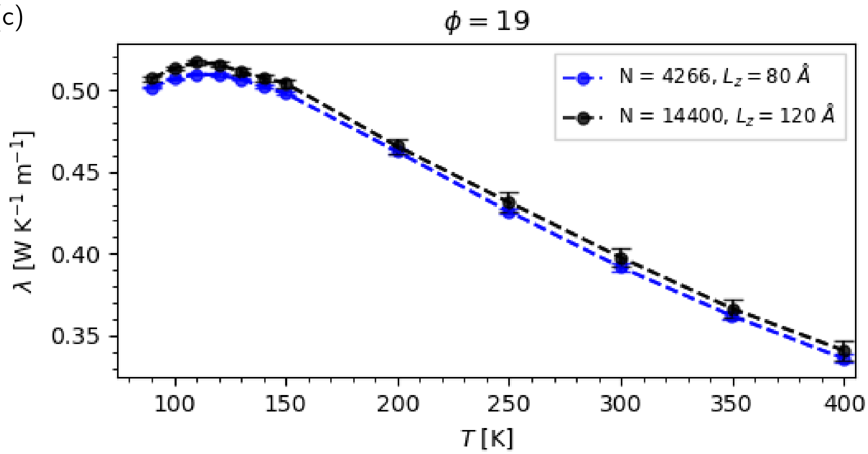

$\phi = 19$
N = 4266, $L_z = 80$ Å
N = 14400, $L_z = 120$ Å
λ [W K^{-1} m^{-1}]
0.50
0.45
0.40
0.35
100
150
200
250
300
350
400
T [K]

(d)

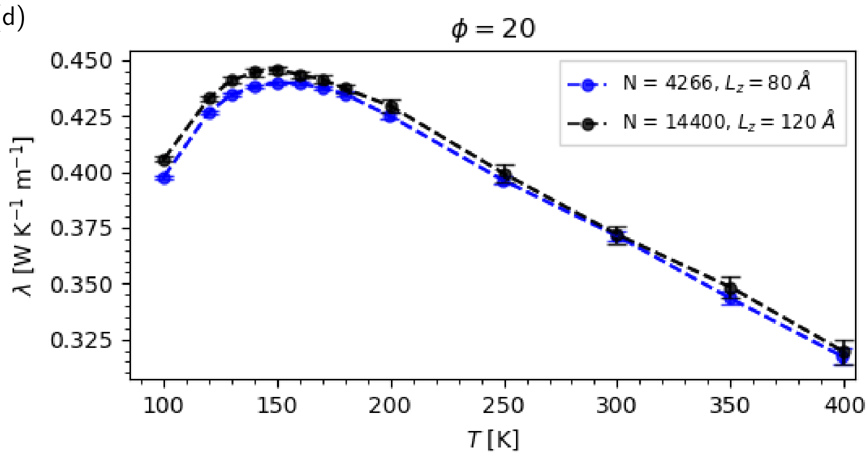

$\phi = 20$
N = 4266, $L_z = 80$ Å
N = 14400, $L_z = 120$ Å
λ [W K^{-1} m^{-1}]
0.450
0.425
0.400
0.375
0.350
0.325
100
150
200
250
300
350
400
T [K]

(e)

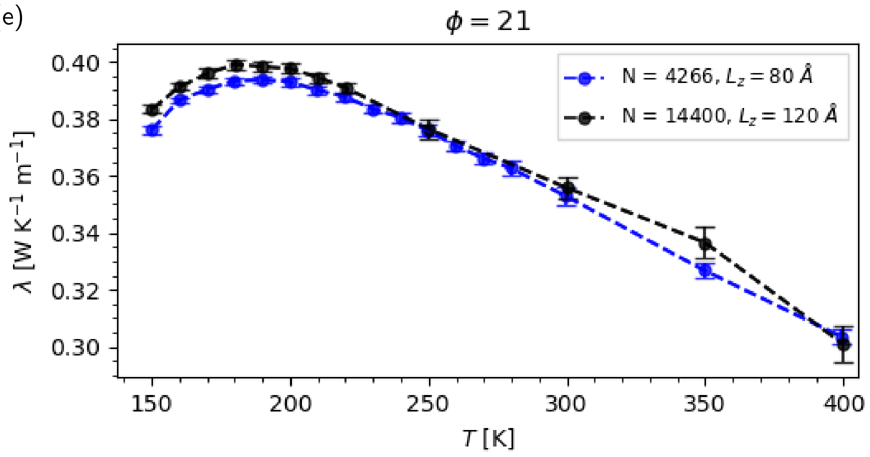
$\phi = 21$
N = 4266, $L_z = 80$ Å
N = 14400, $L_z = 120$ Å
λ [W K^{-1} m^{-1}]
T [K]

(f)

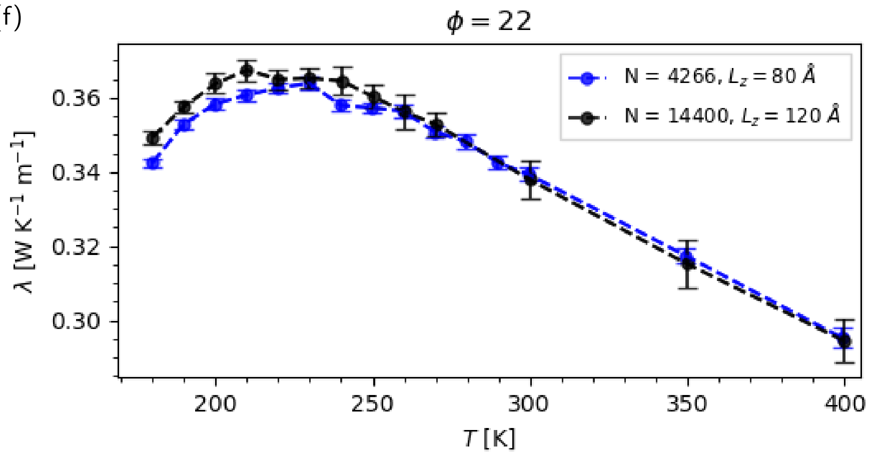
$\phi = 22$
N = 4266, $L_z = 80$ Å
N = 14400, $L_z = 120$ Å
λ [W K^{-1} m^{-1}]
T [K]

(g)

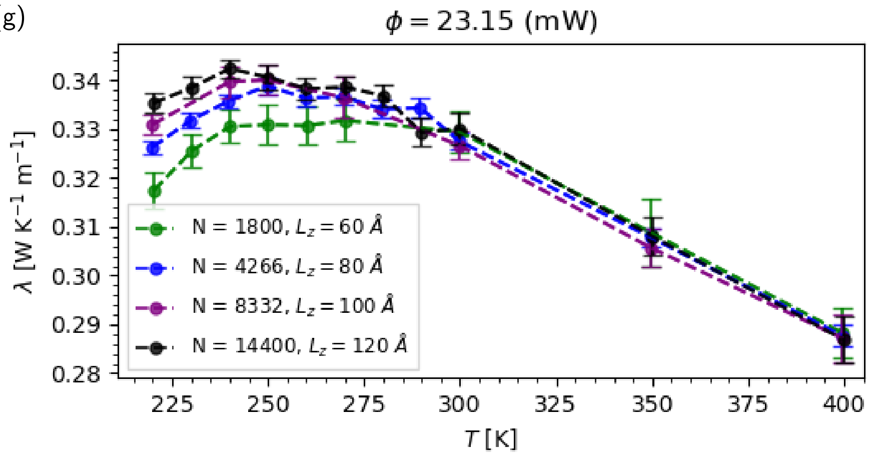
$\phi = 23.15$ (mW)
N = 1800, $L_z = 60$ Å
N = 4266, $L_z = 80$ Å
N = 8332, $L_z = 100$ Å
N = 14400, $L_z = 120$ Å
λ [W K^{-1} m^{-1}]
T [K]

(h)

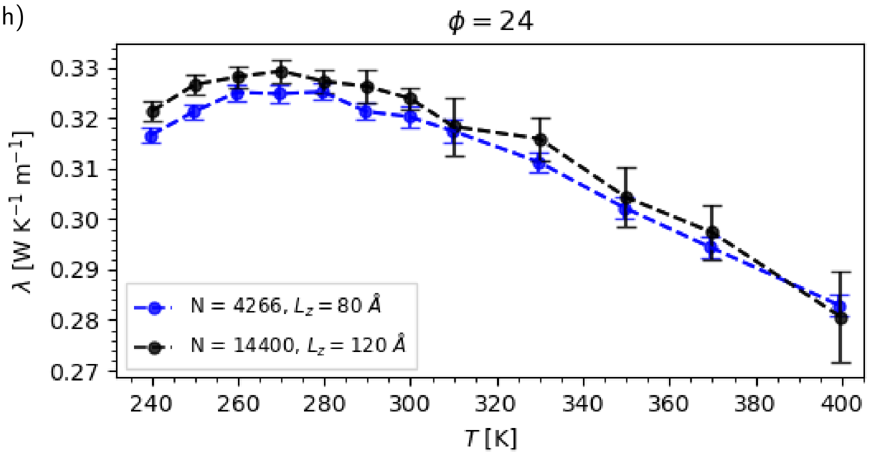
$\phi = 24$
0.33
0.32
0.31
0.30
0.29
0.28
0.27
λ [W K^{-1} m^{-1}]
N = 4266, $L_z = 80$ Å
N = 14400, $L_z = 120$ Å
240
260
280
300
320
340
360
380
400
T [K]

(i)

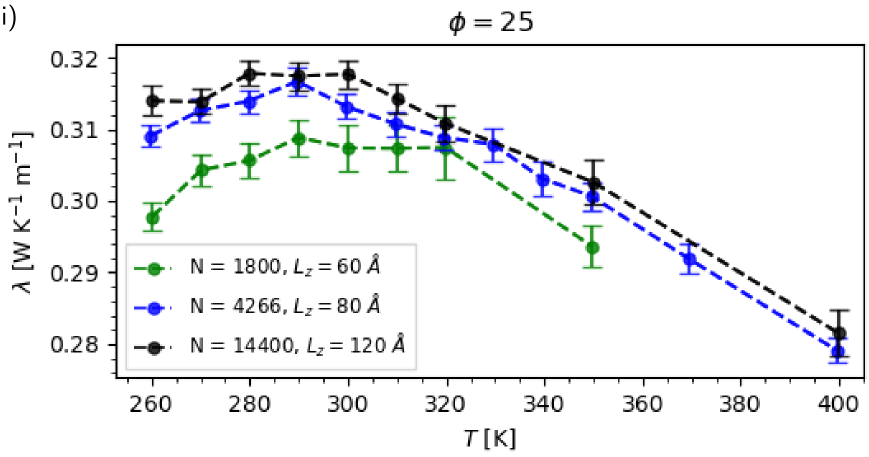
$\phi = 25$
0.32
0.31
0.30
0.29
0.28
λ [W K^{-1} m^{-1}]
N = 1800, $L_z = 60$ Å
N = 4266, $L_z = 80$ Å
N = 14400, $L_z = 120$ Å
260
280
300
320
340
360
380
400
T [K]

(j)

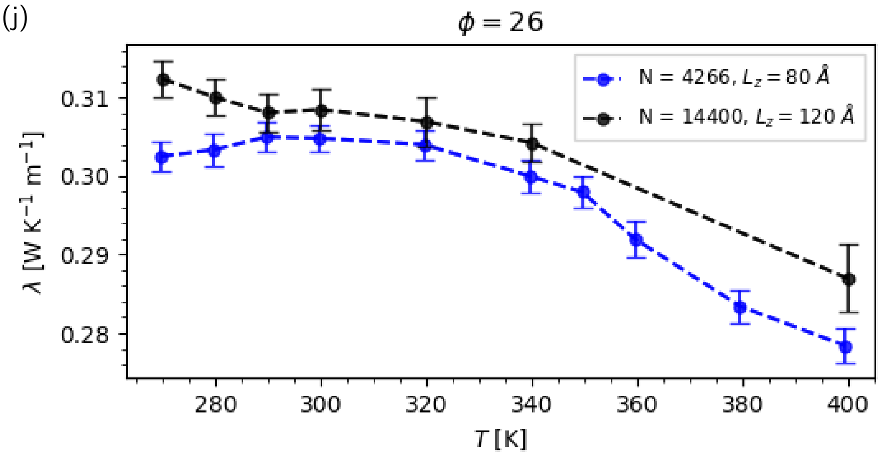
$\phi = 26$
0.31
0.30
0.29
0.28
λ [W K^{-1} m^{-1}]
N = 4266, $L_z = 80$ Å
N = 14400, $L_z = 120$ Å
280
300
320
340
360
380
400
T [K]

(k)

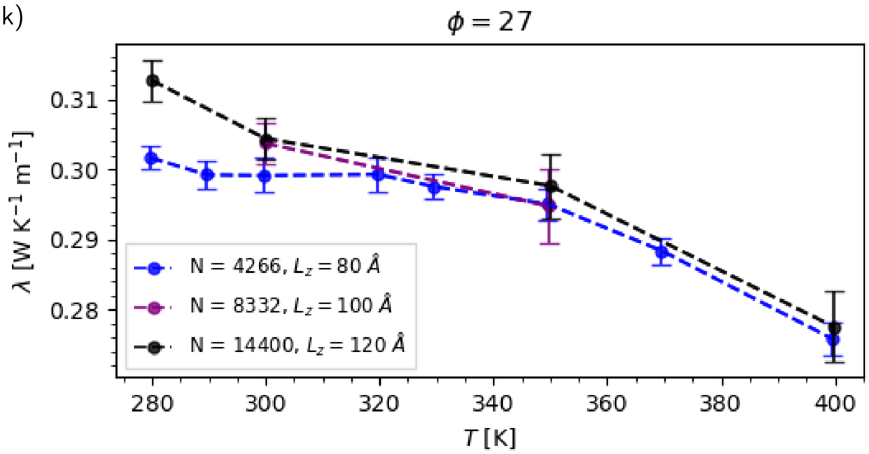

Figure S11: Finite-size convergence of thermal conductivity λ as a function of temperature T for the SW potentials. System size: N is the number of molecules and L_z is the length of the simulation cell in the direction perpendicular to the heat flux. Data corresponds to 10 bar.

2.7.3 Thermal conductivities obtained using very large NEMD simulation cells

The thermal conductivity $\lambda(T)$ can be calculated as a continuous function of temperature T from a single NEMD simulation using Fourier's Law, $\boldsymbol{J}_q = -\lambda(z)\nabla T(z)$, evaluated locally at $T(z)$. This holds so long as ∇T is chosen such that local equilibrium and linear response are fulfilled within the simulation. It follows that $\lambda(T)$ can be calculated over a wide temperature range by increasing increasing L_z while holding $\nabla T \sim$ constant.

However, we observe λ values in large NEMD simulations (Table 3) that are incompatible with the finite-size analysis in section 2.7.2, as shown in Fig. S13(a). This is despite the good agreement between $\rho(T)$ calculated from the NEMD simulations and equilibrium-NPT simulations at the same pressure (Fig. S13(b)), which is a key indicator that local equilibrium is fulfilled. We note that the large NEMD simulations correspond to pressures within $\pm$12 bar of 10 bar; deviations of only $\lesssim 10^{-3}$ W K^{-1} m^{-1} from $\lambda(P/\text{bar} = 10)$ are expected due to the small difference in pressure, which is smaller than the associated uncertainty. Thus, they can be compared with the TC values in section 2.7.2.

In order to investigate the effect of long-range electrostatics (the only long-range potential in our simulations since we use a cutoff for the LJ potential), we compare NEMD simulations that use the long-range P^{3}M Ewald method and the short-range Wolf[39] method. As shown in Fig. S14, the expected finite-size convergence is observed for the Wolf method, suggesting that L_z cannot be increased with a large temperature difference across the simulation cell when using the P^{3}M method as implemented in LAMMPS vs. 7Aug2019 and 29Aug2024.

Table 3: Large NEMD simulation parameters. Symbol meanings are defined in the main SI text.

Force field	N_{mol}	$L_{\parallel}$, L_z [Å]	T_h, T_c [K]	Electro-statics	P [bar]	∇T range [K nm^{-1}]
TIP4P/2005	15,000	31.06, 500	280, 500	P^{3}M	10(1)	8-11
SPC/E	15,000	31.23, 500	280, 500	P^{3}M	12(1)	8-11
SPC	15,000	31.93, 500	280, 500	P^{3}M	0(1)	8-11
TIP3P	15,000	31.93, 500	280, 500	P^{3}M	22(1)	7-12
SPC/E	6,000	31.62, 189.70	280, 465	P^{3}M	4(8)	16-19
SPC/E	30,000	31.62, 948.50	280, 465	P^{3}M	3(5)	3-4
SPC/E	6,000	31.62, 189.70	280, 465	Wolf	218(6)	16-20
SPC/E	30,000	31.62, 948.50	280, 465	Wolf	221(5)	3-4

^{a} CSVR(τ = 100 fs), $N_{\text{rep}} = 30$, $t_{\text{stat}} \geq 1$ ns, t_{prod} =13-14 ns.

^{b} CSVR(τ = 100 fs), $N_{\text{rep}} = 5$, $t_{\text{stat}} \geq 1$ ns, t_{prod} =5 ns.

Other simulation details are the same as the "Rigid" category in Table 1.

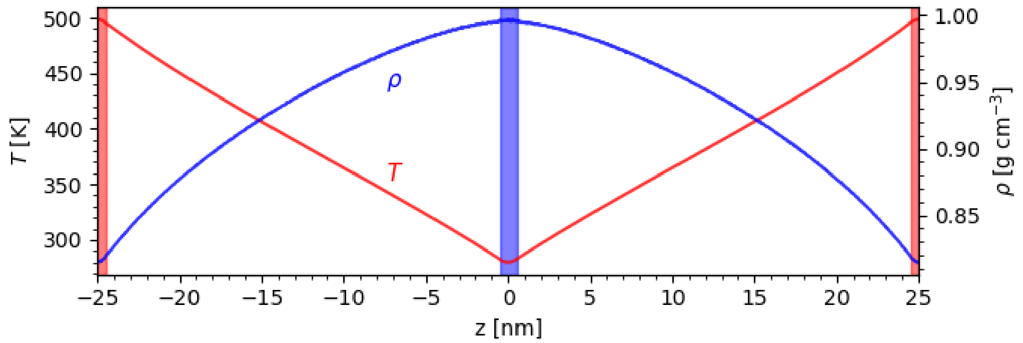

Figure S12: Representative temperature T and density ρ profiles for the large NEMD simulations. The blue (cold) and red (hot) indicate the location of the thermostatting regions in the simulation cell. The profiles correspond to TIP4P/2005 and $P = (10 \pm 1)$ bar.

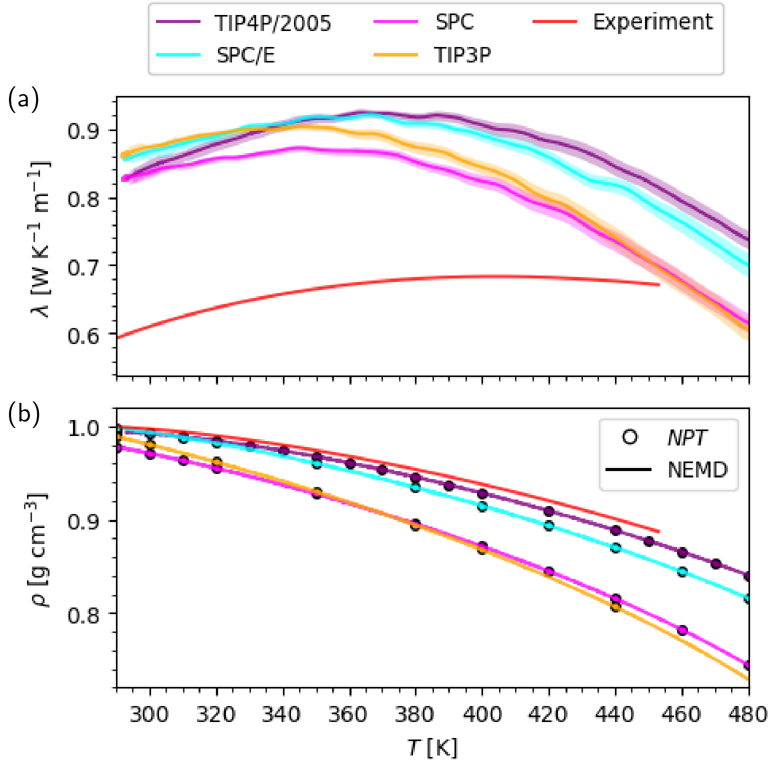

Figure S13: (a) Thermal conductivity λ of selected force fields as a function of temperature T, as calculated from a single large NEMD simulation cell. (b) The density $\rho(T)$ from the NEMD and equilibrium-NPT simulations. Details for each NEMD system are given in Table 3.

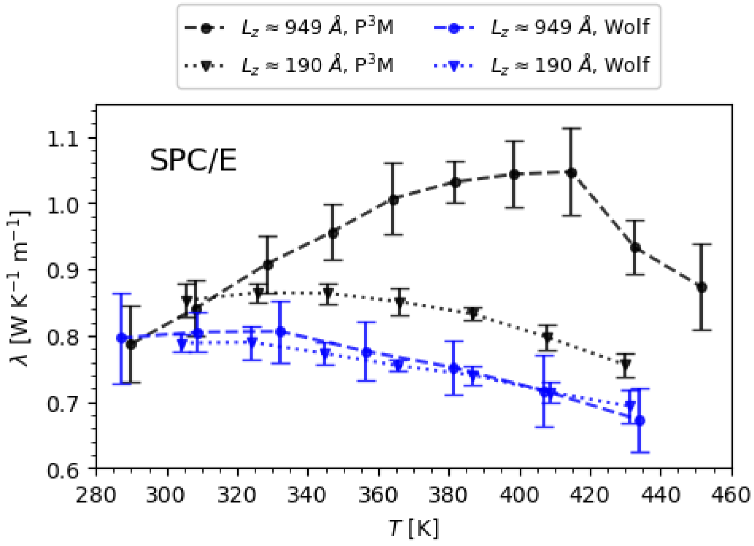

Figure S14: The effect of system size (L_z) and the treatment of electrostatics on the thermal conductivity λ of SPC/E as a function of temperature T, as calculated from a single large NEMD simulation cell. Details for each NEMD system are given in Table 3.

2.8 Radial distribution functions

The figures below show the radial distribution functions for the SW models investigated in this work.

(a)

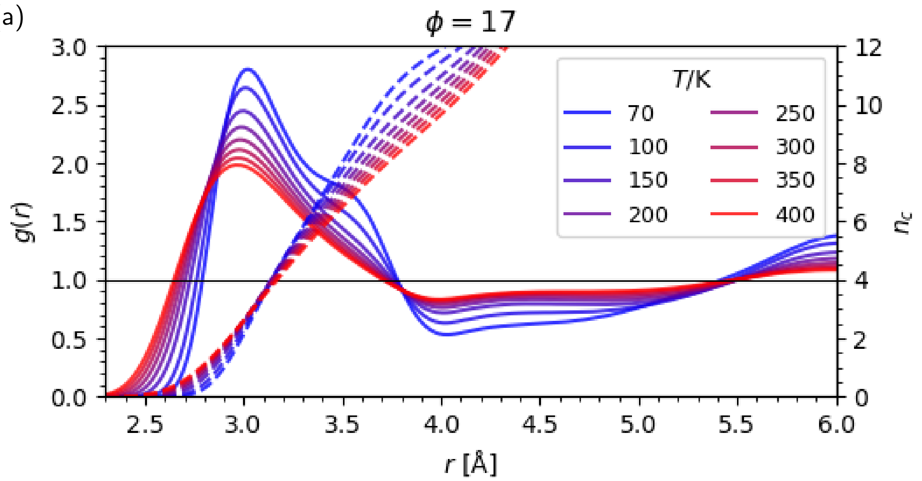

(b)

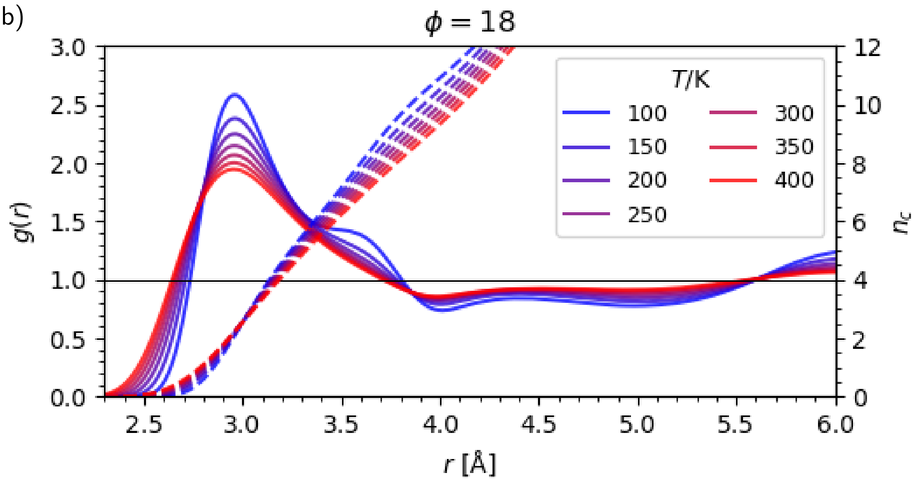

(c)

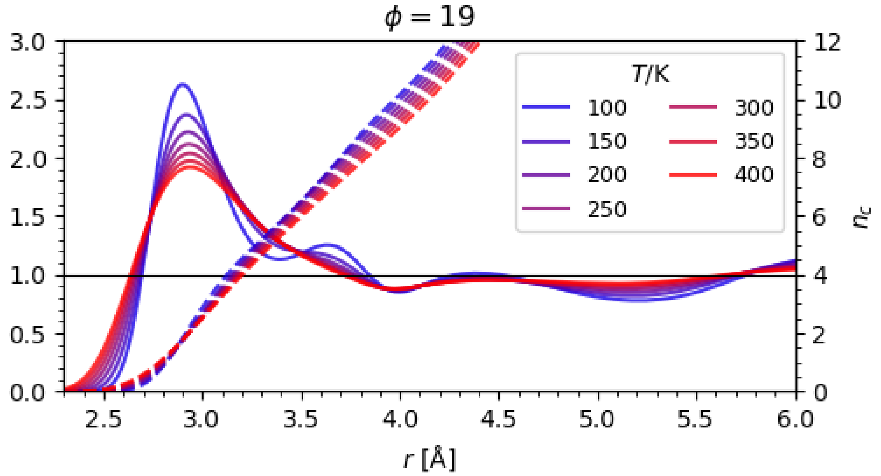
$\phi = 19$
T/K
100
150
200
250
300
350
400
$g(r)$
n_c
r [Å]
3.0 2.5 2.0 1.5 1.0 0.5 0.0
12 10 8 6 4 2 0
2.5 3.0 3.5 4.0 4.5 5.0 5.5 6.0

(d)

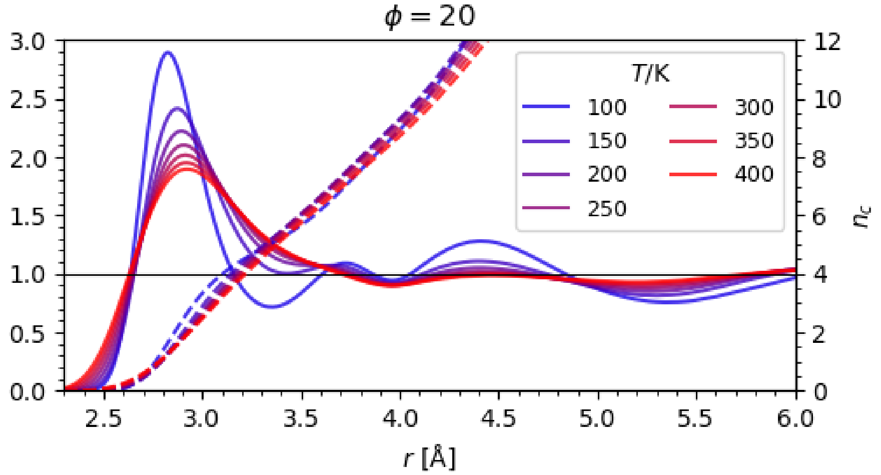
$\phi = 20$
T/K
100
150
200
250
300
350
400
$g(r)$
n_c
r [Å]
3.0 2.5 2.0 1.5 1.0 0.5 0.0
12 10 8 6 4 2 0
2.5 3.0 3.5 4.0 4.5 5.0 5.5 6.0

(e)

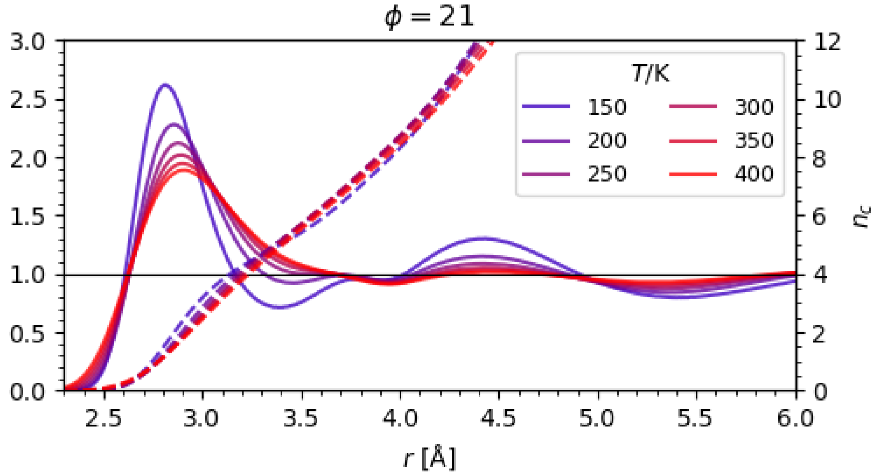
$\phi = 21$
T/K
150
200
250
300
350
400
$g(r)$
n_c
r [Å]
3.0 2.5 2.0 1.5 1.0 0.5 0.0
12 10 8 6 4 2 0
2.5 3.0 3.5 4.0 4.5 5.0 5.5 6.0

(f)

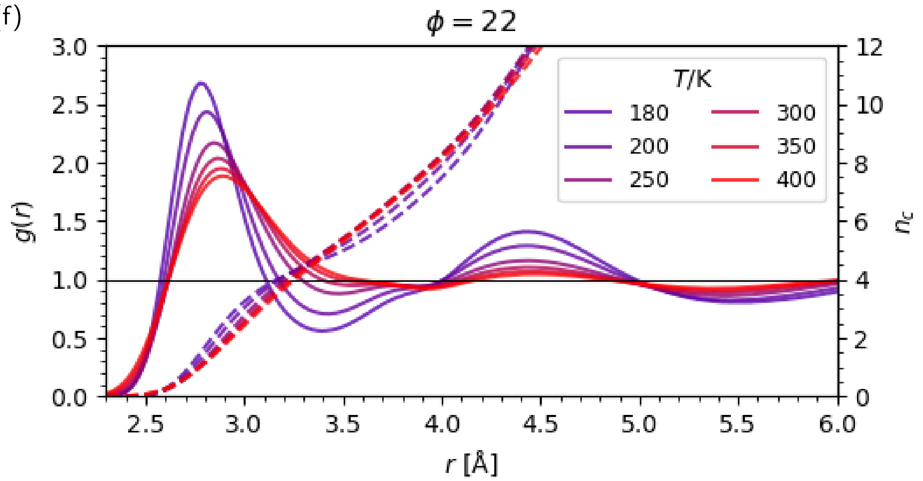
$\phi = 22$
3.0
2.5
2.0
1.5
1.0
0.5
0.0
g(r)
12
10
8
6
4
2
0
n_c
2.5
3.0
3.5
4.0
4.5
5.0
5.5
6.0
r [Å]
T/K
180
200
250
300
350
400

(g)

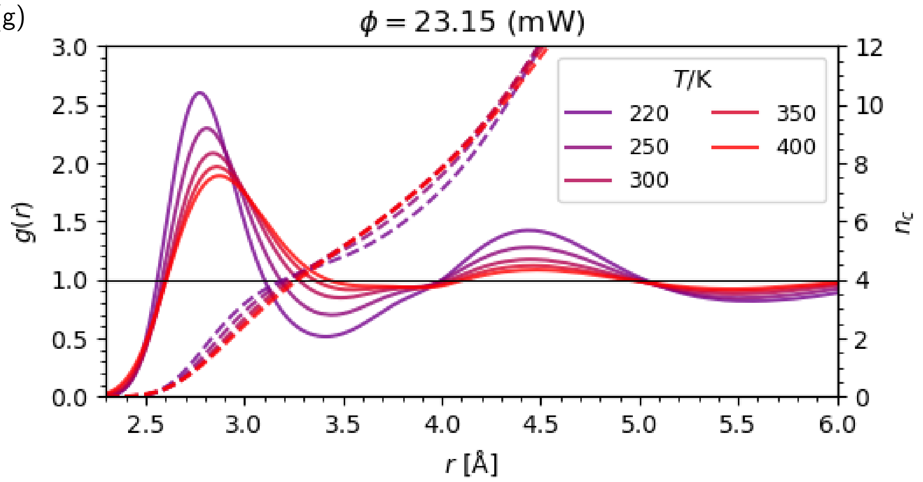
$\phi = 23.15$ (mW)
3.0
2.5
2.0
1.5
1.0
0.5
0.0
g(r)
12
10
8
6
4
2
0
n_c
2.5
3.0
3.5
4.0
4.5
5.0
5.5
6.0
r [Å]
T/K
220
250
300
350
400

(h)

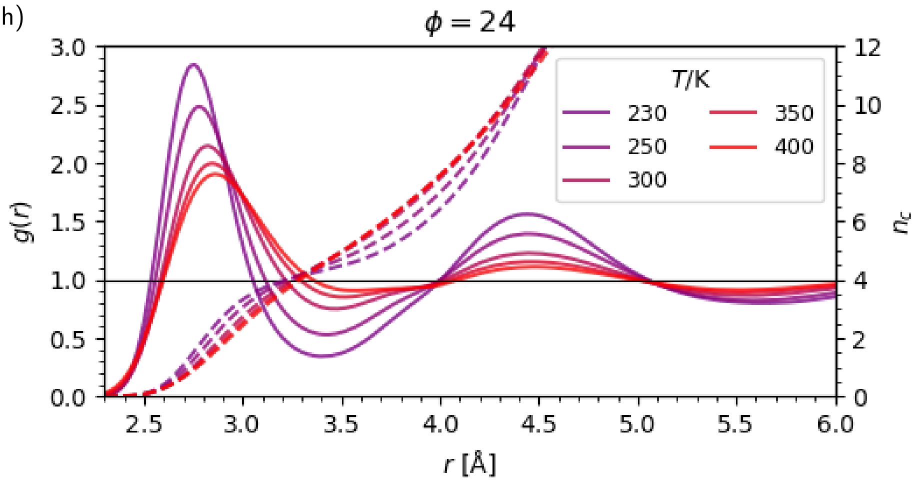
$\phi = 24$
3.0
2.5
2.0
1.5
1.0
0.5
0.0
g(r)
12
10
8
6
4
2
0
n_c
2.5
3.0
3.5
4.0
4.5
5.0
5.5
6.0
r [Å]
T/K
230
250
300
350
400

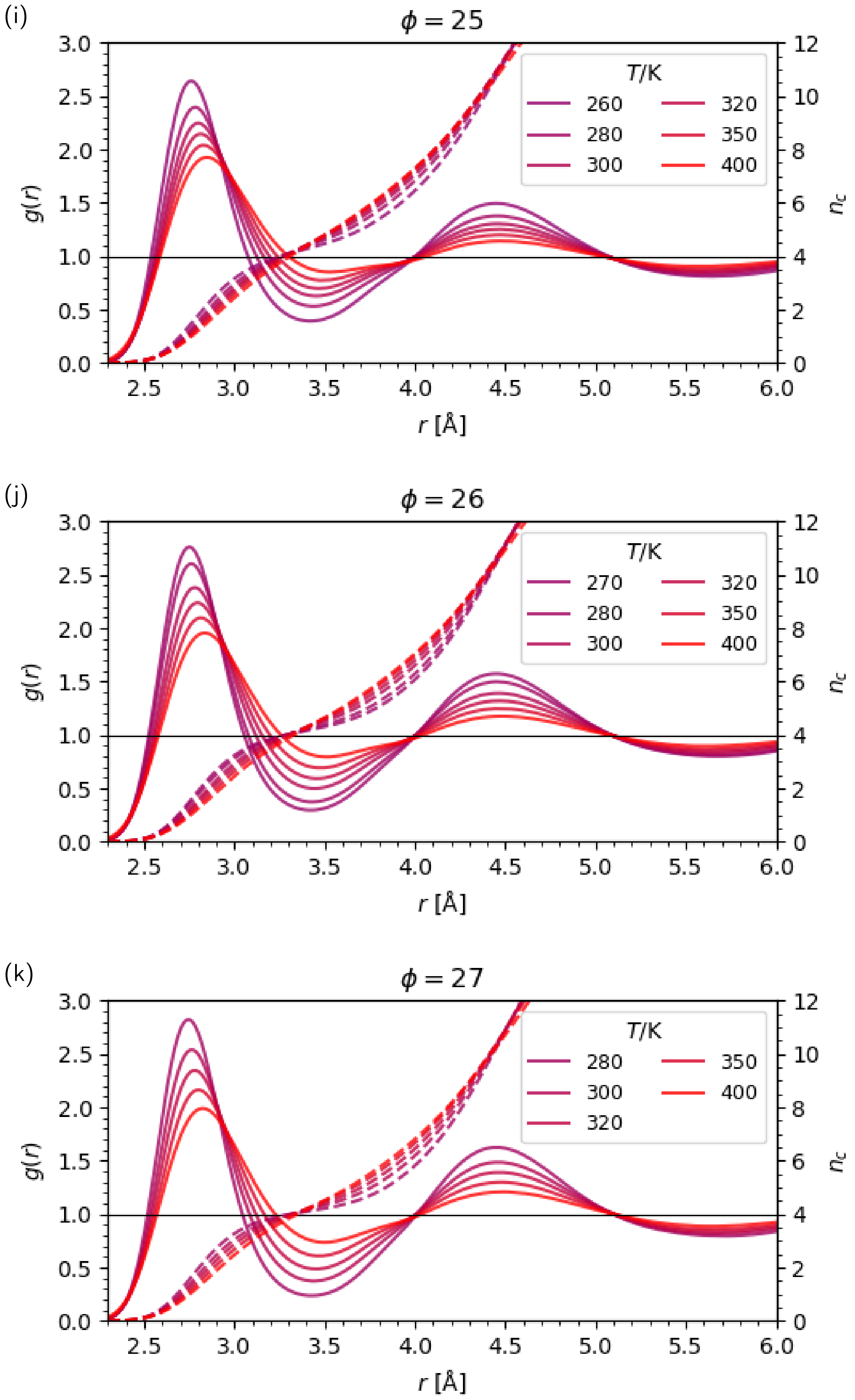

Figure S15: Radial distribution functions $g(r)$ and coordination numbers n_c for the SW potentials at selected temperatures T and 10 bar. r is the radial distance between two particles. The solid lines denote $g(r)$ (left axis), while the dashed lines denote c_n (right axis). The lines are colour coded consistently across all subplots according to the temperature.

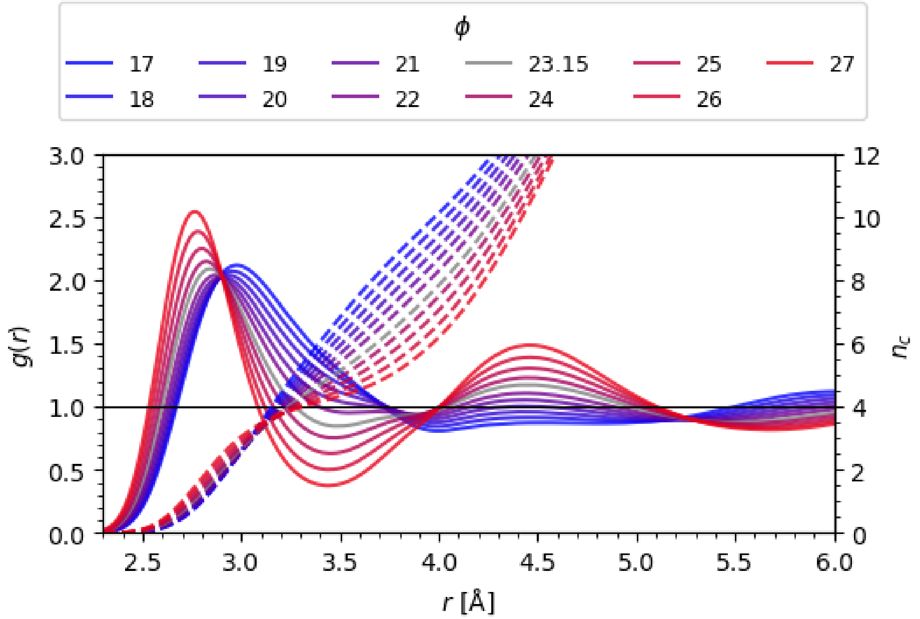

Figure S16: Radial distribution functions $g(r)$ and coordination numbers n_c for the SW potentials at 300 K and 10 bar. r is the radial distance between two particles. The solid lines denote $g(r)$ (left axis), while the dashed lines denote c_n (right axis).

References

[1]A. P. Thompson, H. M. Aktulga, R. Berger, D. S. Bolintineanu, W. M. Brown, P. S. Crozier, P. J. in 't Veld, A. Kohlmeyer, S. G. Moore, T. D. Nguyen, R. Shan, M. J. Stevens, J. Tranchida, C. Trott, and S. J. Plimpton, "LAMMPS - a flexible simulation tool for particle-based materials modeling at the atomic, meso, and continuum scales", Comp. Phys. Comm. **271**, 108171 (2022).

[2]T. F. Miller, M. Eleftheriou, P. Pattnaik, A. Ndirango, D. Newns, and G. J. Martyna, "Symplectic quaternion scheme for biophysical molecular dynamics", J. Chem. Phys. **116**, 8649–8659 (2002).

[3]H. C. Andersen, "Rattle: a "velocity" version of the shake algorithm for molecular dynamics calculations", J. Comput. Phys. **52**, 24–34 (1983).

[4]J.-P. Ryckaert, G. Ciccotti, and H. J. Berendsen, "Numerical integration of the cartesian equations of motion of a system with constraints: molecular dynamics of n-alkanes", J. Comput. Phys. **23**, 327–341 (1977).

[5]M. Allen and D. Tildesley, *Computer Simulation of Liquids* (Oxford: Clarendon Pr, 1987).

[6]E. Pollock and J. Glosli, "Comments on P3M, FMM, and the Ewald method for large periodic Coulombic systems", Comput. Phys. Commun. **95**, 93–110 (1996).

[7]H. M. Aktulga, J. C. Fogart, S. A. Pandit, and A. Y. Grama, "Parallel reactive molecular dynamics: numerical methods and algorithmic techniques", Parallel Comput. **38**, 245–259 (2012).

[8]M. A. González and J. L. F. Abascal, "A flexible model for water based on TIP4P/2005", J. Chem. Phys. **135**, 224516 (2011).

[9]P. Kumar and H. E. Stanley, "Thermal conductivity minimum: a new water anomaly", J. Phys. Chem. B **115**, 14269–14273 (2011).

[10]F. Bresme, J. W. Biddle, J. V. Sengers, and M. A. Anisimov, "Communication: minimum in the thermal conductivity of supercooled water: a computer simulation study", J. Chem. Phys. **140**, 161104 (2014).

[11]G. Zhao and F. Bresme, "Thermal transport and thermal polarization of water in the supercooled regime", J. Phys. Chem. Lett. **15**, 9774–9779 (2024).

[12]C. Vega and J. L. F. Abascal, "Simulating water with rigid non-polarizable models: a general perspective", Phys. Chem. Chem. Phys. **13**, 19663–19688 (2011).

[13]M. L. Huber, R. A. Perkins, D. G. Friend, J. V. Sengers, M. J. Assael, I. N. Metaxa, K. Miyagawa, R. Hellmann, and E. Vogel, "New international formulation for the thermal conductivity of H_2O", J. Phys. Chem. Ref. Data **41**, 033102 (2012).

[14]J. W. Biddle, V. Holten, J. V. Sengers, and M. A. Anisimov, "Thermal conductivity of supercooled water", Phys. Rev. E **87**, 042302 (2013).

[15]W. Wagner and A. Pruß, "The IAPWS formulation 1995 for the thermodynamic properties of ordinary water substance for general and scientific use", J. Phys. Chem. Ref. Data **31**, 387–535 (2002).

[16]P. H. Berens, D. H. J. Mackay, G. M. White, and K. R. Wilson, "Thermodynamics and quantum corrections from molecular dynamics for liquid water", J. Chem. Phys **79**, 2375–2389 (1983).

[17]C. Chapados and J.-J. Max, "Isotope effects in liquid water by infrared spectroscopy. iii. H_2O and D_2O spectra from 6000 to 0 cm^{-1}", J. Chem. Phys. **131**, 184505 (2009).

[18]S. Y. Venyaminov and F. G. Prendergast, "Water (H_2O and D_2O) molar absorptivity in the 1000-4000 cm^{-1} range and quantitative infrared spectroscopy of aqueous solutions", Anal. Biochem. **248**, 234–245 (1997).

[19]D. M. Carey and G. M. Korenowski, "Measurement of the raman spectrum of liquid water", J. Chem. Phys. **108**, 2669–2675 (1998).

[20]G. E. Walrafen, "Raman spectral studies of water structure", J. Chem. Phys. **40**, 3249–3256 (1964).

[21]M. Puligheddu and G. Galli, "Atomistic simulations of the thermal conductivity of liquids", Phys. Rev. Mater. **4**, 053801 (2020).

[22] O. R. Gittus and F. Bresme, “Thermophysical properties of water using reactive force fields”, J. Chem. Phys. **155**, 114501 (2021).

[23] T. Ohara, “Intermolecular energy transfer in liquid water and its contribution to heat conduction: a molecular dynamics study”, J. Chem. Phys. **111**, 6492–6500 (1999).

[24] D. Bedrov and G. D. Smith, “Thermal conductivity of molecular fluids from molecular dynamics simulations: application of a new imposed-flux method”, J. Chem. Phys. **113**, 8080–8084 (2000).

[25] F. Bresme, “Equilibrium and nonequilibrium molecular-dynamics simulations of the central force model of water”, J. Chem. Phys. **115**, 7564–7574 (2001).

[26] M. Zhang, E. Lussetti, L. E. S. de Souza, and F. Müller-Plathe, “Thermal conductivities of molecular liquids by reverse nonequilibrium molecular dynamics”, J. Phys. Chem. B **109**, 15060–15067 (2005).

[27] T. Terao and F. Müller-Plathe, “A nonequilibrium molecular dynamics method for thermal conductivities based on thermal noise”, J. Chem. Phys. **122**, 081103 (2005).

[28] W. Evans, J. Fish, and P. Keblinski, “Thermal conductivity of ordered molecular water”, J. Chem. Phys. **126**, 154504 (2007).

[29] E. J. Rosenbaum, N. J. English, J. K. Johnson, D. W. Shaw, and R. P. Warzinski, “Thermal conductivity of methane hydrate from experiment and molecular simulation”, J. Phys. Chem. B **111**, 13194–13205 (2007).

[30] H. Jiang, E. M. Myshakin, K. D. Jordan, and R. P. Warzinski, “Molecular dynamics simulations of the thermal conductivity of methane hydrate”, J. Phys. Chem. B **112**, 10207–10216 (2008).

[31] S. Kuang and J. D. Gezelter, “A gentler approach to rnemd: nonisotropic velocity scaling for computing thermal conductivity and shear viscosity”, J. Chem. Phys. **133**, 164101 (2010).

[32] J. Muscatello and F. Bresme, “A comparison of coulombic interaction methods in non-equilibrium studies of heat transfer in water”, J. Chem. Phys. **135**, 234111 (2011).

[33] J. Muscatello, F. Römer, J. Sala, and F. Bresme, “Water under temperature gradients: polarization effects and microscopic mechanisms of heat transfer”, Phys. Chem. Chem. Phys. **13**, 19970–19978 (2011).

[34] F. Römer, A. Lervik, and F. Bresme, “Nonequilibrium molecular dynamics simulations of the thermal conductivity of water: a systematic investigation of the SPC/E and TIP4P/2005 models”, J. Chem. Phys. **137**, 074503 (2012).

[35] Y. Mao and Y. Zhang, “Thermal conductivity, shear viscosity and specific heat of rigid water models”, Chem. Phys. Lett. **542**, 37–41 (2012).

[36]T. W. Sirk, S. Moore, and E. F. Brown, "Characteristics of thermal conductivity in classical water models", J. Chem. Phys. **138**, 064505 (2013).

[37]S. H. Lee, "Temperature dependence of the thermal conductivity of water: a molecular dynamics simulation study using the SPC/E model", Mol. Phys. **112**, 2155–2159 (2014).

[38]S. H. Lee and J. Kim, "Transport properties of bulk water at 243–550 K: a comparative molecular dynamics simulation study using SPC/E, TIP4P, and TIP4P/2005 water models", Mol. Phys. **117**, 1926–1933 (2019).

[39]D. Wolf, P. Keblinski, S. R. Phillpot, and J. Eggebrecht, "Exact method for the simulation of coulombic systems by spherically truncated, pairwise r^{-1} summation", J. Chem. Phys. **110**, 8254–8282 (1999).
